# Circular RNA MARCH3 promotes influenza A virus replication by coordinating NF-κB negative feedback and the miR-3657/ATG7 axis

**DOI:** 10.3389/fimmu.2026.1948323

**Published:** 2026-10-22

**Authors:** Jiayin Liu, Jiarong Song, Ling’en Yang, Mohamed Maarouf, Haori Qiu, Shasha Liu, Shile Huang, Song Wang, Ji-Long Chen

**Affiliations:** 1 Key Laboratory of Animal Pathogen Infection and Immunology of Fujian Province, College of Animal Science, Fujian Agriculture and Forestry University, Fuzhou, China; 2 Key Laboratory of Fujian-Taiwan Animal Pathogen Biology, College of Animal Science, Fujian Agriculture and Forestry University, Fuzhou, China; 3 Joint Laboratory of Animal Pathogen Prevention and Control of Fujian-Nepal, College of Animal Science, Fujian Agriculture and Forestry University, Fuzhou, China; 4 Department of Biochemistry and Molecular Biology, Louisiana State University Health Sciences Center, Shreveport, LA, United States

**Keywords:** circMARCH3, influenza A virus, innate immunity, miR-3657, NF-κB

## Abstract

**Introduction:**

Circular RNAs (circRNAs) have emerged as important regulators of diverse cellular processes, and accumulating evidence indicates that certain circRNAs modulate immune responses through complex mechanisms. However, their roles in innate immunity against influenza A virus (IAV) infection remain poorly understood.

**Methods:**

We identified a previously uncharacterized circRNA, termed circMARCH3, through RNA-seq, RNase R digestion assay, and RNA stability assay. We examined circMARCH3 expression in multiple cell types following viral infection by RT-PCR and real-time PCR, investigated the signaling pathway driving its induction, and performed functional analyses using circMARCH3 overexpression vectors and shRNAs-mediated knockdown. Furthermore, we identified and validated the circMARCH3/miR-3657/ATG7 axis through mechanistic studies.

**Results:**

circMARCH3 is generated from the pre-mRNA of the membrane-associated RING-CH-Type Finger 3 (MARCH3) gene, and its expression was robustly induced by IAV and several other viral infections in multiple cell types. Mechanistically, virus-induced circMARCH3 expression was driven by the NF-κB signaling pathway. Functional analyses demonstrated that circMARCH3 overexpression significantly promoted IAV replication, whereas circMARCH3 knockdown markedly inhibited viral production. Consistent with these findings, circMARCH3 functioned as a negative regulator of antiviral innate immunity by attenuating NF-κB activation and type I interferon responses. Furthermore, we identified that circMARCH3 targeted miR-3657, a microRNA that suppresses the expression of ATG7, a key autophagy regulator and known pro-IAV factor. Mechanistic studies revealed that circMARCH3 acted as a competitive endogenous RNA (ceRNA), sequestering miR-3657 and thereby relieving its repressive effect on ATG7 expression, resulting in enhanced autophagy and increased viral replication.

**Discussion:**

The results demonstrate circMARCH3 as a virus-inducible circRNA that promotes IAV infection through two complementary mechanisms: dampening NF-κB-mediated antiviral innate immunity and facilitating autophagy via the circMARCH3/miR-3657/ATG7 axis. Our findings establish circMARCH3 as a novel regulator of the complex interaction between host and IAV by coordinating NF-κB negative feedback and the miR-3657/ATG7 axis.

## 1 Introduction

Influenza A virus (IAV) causes infectious respiratory diseases in humans and various animals, resulting in numerous human deaths worldwide annually and huge economic losses in the animal breeding industry (1–3). As a member of the *Orthomyxoviridae* family, IAV is a negative-sense single-stranded RNA virus containing eight negative-sense segments in its genome, which encode 12 viral proteins (4). IAV exhibits a high propensity for antigenic drift and antigenic shift, enabling it to evade host antiviral immunity and complicating vaccine development (5–7). The virus is classified into numerous subtypes due to potential combinations of 16 hemagglutinin (HA) and 9 neuraminidase (NA) (8). Among these, several subtypes of IAV, such as H1N1, H2N2, and H3N2, still pose a significant threat to human health (9–11). Although progress has been made in elucidating the interaction between IAV and the host, the pathogenesis of IAV is not fully understood.

Increasing evidence indicates that non-coding RNAs are important in the regulation of IAV pathogenesis (12, 13). CircRNAs are a distinctive class of non-coding RNA molecules characterized by a covalently closed circular structure. Such circular structure is formed from pre-mRNA through a process called “back-splicing” (12). The back-splicing results in circRNAs lacking a 5′N7-methylguanosine (m7G) cap and a 3′ poly(A) tail, which makes circRNAs more conserved and more stable than their linear counterparts (13, 14). CircRNAs have been implicated in various physiological processes, including cell proliferation, development, metabolism, aging, and regeneration (15–19). Moreover, they are involved in pathological conditions such as tumorigenesis, heart diseases, and neurodegenerative disorders (20–22). Recently, a growing number of studies have indicated that circRNAs play critical roles in these processes via a wide range of mechanisms, such as regulating transcription and splicing, binding to RNA-binding proteins, acting as sponges for microRNA (miRNA), and encoding functional microproteins (23–26). However, many circRNAs likely remain unidentified.

It is well known that some non-coding RNAs act as a competitive endogenous RNA (ceRNA) to regulate certain mRNA expression (27–29). These RNAs can interact with each other through shared miRNA binding sites, forming an extensive regulatory network. In this network, miRNAs and transcripts from protein-coding genes, pseudogenes, and long non-coding RNAs are central components (30, 31). Through ceRNA network, long non-coding RNAs, miRNAs, and target mRNAs can actively communicate with each other to regulate their expression levels. This communication is mediated by the miRNA response elements (MREs), which are fundamental to the ceRNA network (32). RNAs that share multiple MREs can engage in effective crosstalk, forming a vast and multifunctional regulatory network (30). The functionality and efficiency of a ceRNA network depend on several prerequisites, including changes in ceRNA expression and subcellular localization, the abundance of miRNAs that a ceRNA can “sponge”, and the number of target sites per miRNA (33). The ceRNA research has significantly expanded our understanding of non-coding RNAs function. As members of the non-coding RNA family, circRNAs exhibit considerable potential to function as ceRNAs, although the precise mechanisms remain to be determined.

The membrane-associated RING-CH-type finger (MARCH) proteins are E3 ubiquitin ligases. MARCH proteins were first identified as candidates for the cellular origin of viral K3 and K5, which are RING-CH-containing E3 ligases encoded by Kaposi’s sarcoma-associated herpesvirus (KSHV) (34, 35). MARCH proteins are located in different cellular compartments, including endosomes, lysosomes, endoplasmic reticulum (ER), Golgi apparatus, cytoplasm and plasma membrane, and their subcellular distribution directly influences their function (36). Among them, MARCH3 is highly expressed in the spleen, colon, and lung, and acts as a negative regulator of inflammatory signaling (37). It attenuates interleukin-3 (IL-3)-induced responses by mediating K48-linked polyubiquitination and degradation of IL-3 receptor alpha (IL-3Rα) (38). Similarly, MARCH3 suppresses IL-1β-triggered signal transduction by inhibiting nuclear factor kappa B (NF-κB) activation and the expression of inflammatory genes (39). It also targets IL-5 receptor alpha (IL-5Rα) at lysine 383 for K27-linked polyubiquitination, thereby negatively regulating IL-5-mediated signaling (40). While the roles of MARCH1, MARCH4, and MARCH9 have been documented to be involved in immune responses, little information is available about the functional relevance of MARCH3 in antiviral responses (41–43). Notably, it remains unclear whether there is a circRNA derived from back-splicing of MARCH3 pre-mRNA.

Previous studies from our group and others have revealed the functional involvement of circRNAs, such as circMYO9A, circMerTK and circRNA_0050463 in the IAV infection (44–46). However, the roles and underlying mechanisms of circRNAs in IAV-host interactions are still largely unknown. In this study, we identified a new circRNA derived from pre-mRNA of MARCH3, called circMARCH3. We observed that circMARCH3 is significantly upregulated by IAV and several other viral infections in host cells. Further studies revealed that circMARCH3 promotes influenza virus infection by two mechanisms: an NF-κB/circMARCH3 negative feedback loop that dampens antiviral innate immunity and a circMARCH3/miR-3657/ATG7 regulatory axis that enhances autophagy. Collectively, our findings uncover a previously unrecognized role for circMARCH3 in promoting IAV infection and provide broader insights into the functions of circRNAs in host-virus interactions.

## 2 Materials and methods

### 2.1 Viruses, viral infection and viral titration

The influenza A virus A/Puerto Rico/8/34 (H1N1) (PR8), A/WSN/1933 (H1N1) (WSN), A/California/04/2009 (H1N1) (CA/04), H9N2, H3N2, and Sendai virus (SeV) were proliferated in specific pathogen-free (SPF) chicken embryos. Pseudorabies virus (PRV) was propagated in PK-15 cells. Muscovy Duck Reovirus (MDRV) was propagated in 293T cells, and Herpes simplex virus (HSV) in Vero cells. For viral infection, monolayer cells of respective cell lines were infected with indicated viruses at a specified multiplicity of infection (MOI), as described by Chi et al. (47). For hemagglutination (HA) assay, supernatants from virus-infected cells were incubated in phosphate-buffered saline (PBS) and mixed with an equal volume of 1% chicken red blood cell. The HA titer was determined according to the end point hemagglutination test. For plaque forming assay (PFA), MDCK cells were infected with serial dilution of virus-infected cell supernatant (37 °C, 1 h), washed with PBS, and covered with α-MEM containing 3% agarose and 2 μg/mL TPCK-trypsin. The resulting plaques were stained with 1% crystal violet and then plaque forming unit (PFU) was counted.

### 2.2 Cell lines and cell culture

The A549, 293T, NIH/3T3, and MDCK cell lines were obtained from American Type Culture Collection (ATCC) (Manassas, VA, USA). All cells were cultured in Dulbecco’s modified Eagle’s medium (DMEM) (Gibco, Grand Island, NY, USA) supplemented with 10% fetal bovine serum (Vazyme, Nanjing, China) and 100 U/mL penicillin-streptomycin (Beyotime Biotechnology, Shanghai, China) at 37 °C under a humidified 5% CO_2_ atmosphere.

### 2.3 RNA extraction, RT-PCR, and qRT-PCR

Total RNA was extracted from cells using TRIzol reagent (Tiangen, Beijing, China). Total RNA (1 μg) was reverse-transcribed into cDNA using HiScript III 1st Strand cDNA Synthesis Kit (Vazyme, Nanjing, China), followed by PCR using Taq DNA polymerase (GenStar, Beijing, China) or quantitative real-time PCR (qRT-PCR) using SYBR Green Master Mix (Vazyme, Nanjing, China). The primer sequences used in this study are listed in **Supplementary Table S1**.

### 2.4 RNA-seq analysis

RNA sequencing (RNA-seq) was performed in our laboratory on lung tissues from IAV PR8-infected and mock-infected mice as described previously by Qiu et al. (45). The raw RNA-seq data have been deposited in the Gene Expression Omnibus (GEO, https://www.ncbi.nlm.nih.gov/geo/) under accession number GSE210688. For miRNA-seq, 293T cells stably expressing control vector (pLC5-ciR) or circMARCH3 (pLC5-circMARCH3) were infected with IAV PR8 for 12 h. Total RNA was extracted with TRIzol reagent, and bioinformatic analysis was completed by Wuhan SeqHealth Co., Ltd. (Wuhan, China). Raw miRNA-seq data were submitted to the NCBI Sequence Read Archive (SRA) under BioProject accession number PRJNA1456839. For miRNA prediction and expression analysis, raw sequencing data were filtered by fastp (v0.23.2). Clean reads were further treated with in-house scripts to eliminate duplication bias. Known and *de novo* predicted miRNAs were identified and quantified using mirDeep2 (version 2.0.1.3). Differentially expressed miRNAs between groups were identified using the edgeR package (version 3.40.2), with a *p*-value cutoff < 0.05 and a fold-change cutoff > 2. Classification and annotation of small RNAs (sRNAs) were performed using RepeatMasker (version 4.0.5). Potential target genes of miRNAs were predicted by miRanda software (version 3.3a).

### 2.5 Dual-luciferase reporter assay

Dual-luciferase reporter assay was carried out as described below. Briefly, 293T cells were seeded into 24-well culture plates. Luciferase reporter of NF-κB or interferon-β (IFN-β) was co-transfected with pRL-TK Renilla luciferase control vector (MiaoLing Plasmid Platform, Wuhan, China), along with pLC5-ciR or pLC5-circMARCH3, using Lipo8000 (Beyotime Institute of Biotechnology, Jiangsu, China). At 24 h post-transfection, luciferase activity was measured using the dual-luciferase reporter assay system (Promega, WI, USA) according to the manufacturer’s instructions.

### 2.6 Cell stimulation

Recombinant human IFN-β and IL-6 were purchased from PeproTech (Cranbury, NJ, USA). polyinosinic-polycytidylic acid [poly(I:C)], BAY 11-7082, lipopolysaccharide (LPS), and dimethyl sulfoxide (DMSO) were purchased from Sigma-Aldrich (St. Louis, MO, USA). For viral RNA and cellular RNA treatment, total RNA was extracted from mock-infected or IAV PR8-infected A549 or NIH/3T3 cells using TRIzol reagent. PR8 genomic RNA (VG-RNA) was extracted from the purified virus particles using viral RNA kit (Omega Bio-tek, Norcross, GA, USA). A549 cells were then transfected with indicated RNA or poly(I:C) using Lipofectamine 3000 (Invitrogen, Carlsbad, CA, USA) for 8 h. For cytokine or LPS stimulation, A549 cells were incubated with recombinant IFN-β, LPS, or IL-6 for 6 h, respectively.

### 2.7 Western blotting

Western blotting was performed as described previously (48). Briefly, cells were lysed with radio immunoprecipitation assay (RIPA) buffer supplemented with protease inhibitors. Cell lysates were separated by SDS-PAGE, transferred onto nitrocellulose membrane, and probed with indicated antibodies.

### 2.8 RNase R digestion assay

Total RNA was extracted from cells using TRIzol reagent. 2 μg of total RNA was incubated with or without 3 U RNase R (Epicentre) at 37 °C for 30 min. The reaction was terminated by incubation at 70 °C for 10 min. The RNA was then purified and reverse-transcribed into cDNA, and the relative expression levels of circMARCH3 and linear MARCH3 mRNA were determined by qRT-PCR.

### 2.9 RNA stability assay with actinomycin D

A549 cells were treated with actinomycin D (5 μg/mL) to block *de novo* RNA transcription and harvested at the indicated time points after the treatment. Total RNA was extracted, and RT-PCR was performed to determine the remaining levels of circMARCH3 and linear MARCH3 mRNA.

### 2.10 RNA immunoprecipitation assay

Cell lysates were incubated with an anti-p65 antibody or control IgG overnight at 4 °C. RNA–protein complexes were captured using protein A/G agarose beads. After washing and purification, co-precipitated RNAs were extracted and subjected to qPCR analysis to determine the enrichment of circMARCH3 and GAPDH mRNA (used as a negative control).

### 2.11 Biotin-labeled miRNA pull-down assay

A biotin pull-down assay was performed to confirm the interaction between miR-3657 and circMARCH3. Briefly, a biotinylated miR-3657 mimics and a negative control were transfected into A549 cells. Cell lysates were then incubated with streptavidin magnetic beads overnight at 4 °C to capture the biotin-labeled miRNA–RNA complexes. After extensive washing, the bound RNAs were eluted and purified, and the enrichment of circMARCH3 was determined by qRT-PCR.

### 2.12 Statistical analysis

Data represent the mean ± SD from three independent experiments. Statistical analysis was performed using Student’s *t* test or one-way ANOVA test. Differences were considered statistically significant with *p* < 0.05.

## 3 Results

### 3.1 CircMARCH3 is identified as a new circRNA induced by IAV infection

To investigate the regulatory roles of circRNAs during IAV infection, our group previously performed RNA-seq on lung tissues from mock- and IAV-infected mice and screened for differentially expressed circRNAs (45). Based on their significant expression changes upon IAV infection, we selected three circRNAs, circMARCH3, circCrk, and circYes1 for further research (**Figure 1A**). To confirm the expression of these circRNAs in human and murine cells, we performed RT-PCR using primers specifically designed to target each circRNA. The results indicated that only circMARCH3 was consistently upregulated in NIH/3T3, A549, and 293T cells infected with IAV strain PR8, whereas circCrk and circYes1 showed inconsistent expression patterns (**Figures 1B–D**). Therefore, our subsequent studies were focused on circMARCH3.

**Figure 1:**
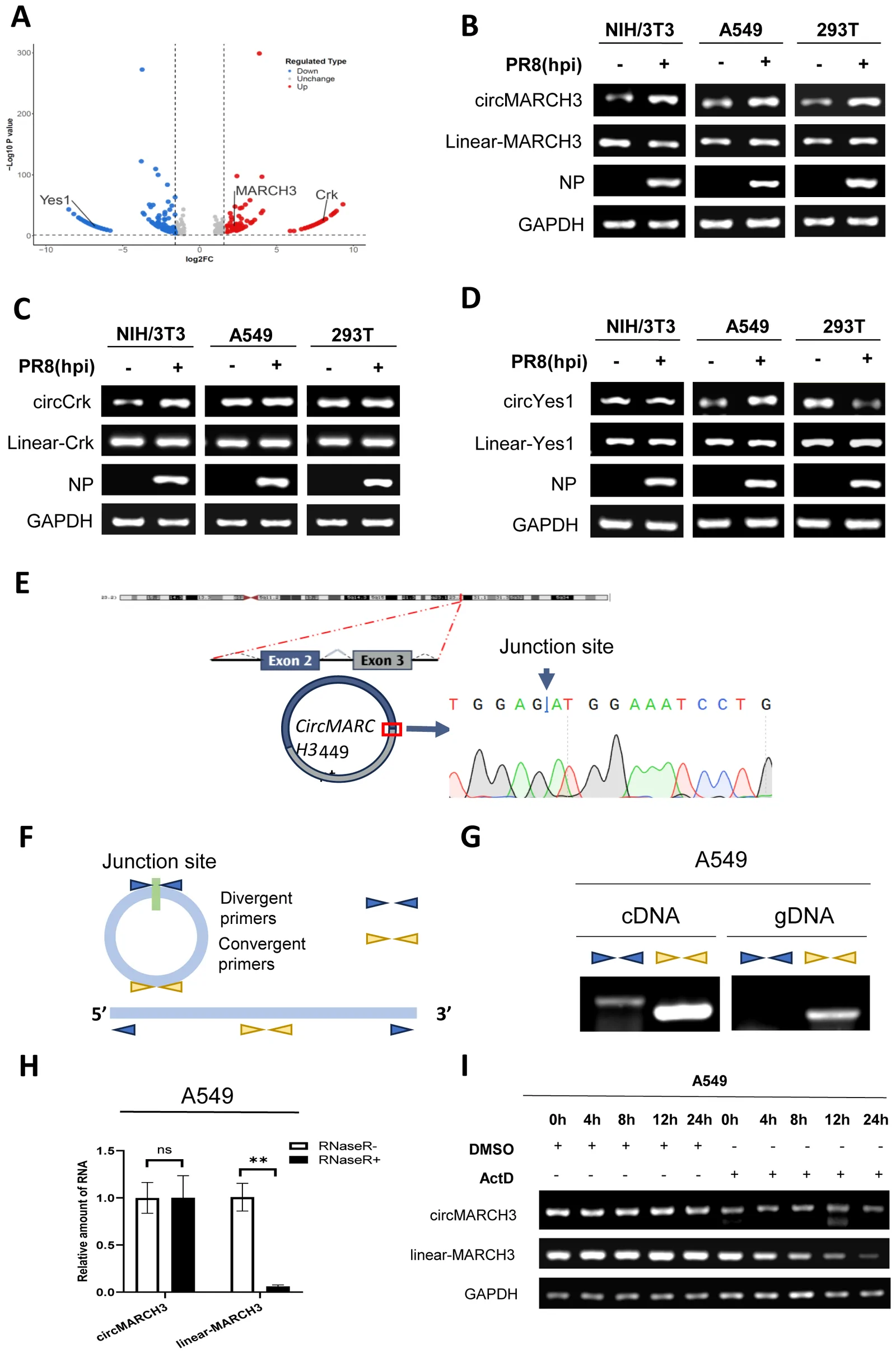
CircMARCH3 is identified as an IAV-induced circRNA. **(A)** Volcano plot comparing circRNAs expression between the IAV-infected group and the mock-infected group. The circRNAs marked in red are upregulated in mouse lung tissues after IAV infection, whereas those marked in blue are downregulated. Differential analysis was performed using the R package DESeq2, with *p*-values adjusted for multiple testing using the false discovery rate (FDR) method. **(B–D)** NIH/3T3, A549, and 293T cells were infected with or without PR8 (MOI = 1) for 16 h. Expression of circMARCH3 **(B)**, circCrk **(C)**, circYes1 **(D)**, and their linear mRNA, together with IAV NP in these cell lines was assessed by RT-PCR, respectively. Membrane-associated RING-CH-type finger 3, MARCH3; CRK proto-oncogene, Crk; YES proto-oncogene 1, Yes1; IAV nucleoprotein, IAV-NP. **(E)** Schematic diagram of circMARCH3 formed by back-splicing from the mouse MARCH3 gene at chromosome 18. **(F)** The schematic diagram of divergent primers and convergent primers. **(G)** Divergent primers and convergent primers amplify results of circMARCH3 in cDNA and gDNA samples. **(H)** Total RNA from A549 cells was incubated with or without RNase R at 37 °C for 30 min, and the relative expression levels of circMARCH3 and linear MARCH3 mRNA were determined by qRT-PCR. **(I)** A549 cells were treated with actinomycin D (5 μg/mL), and the remaining levels of circMARCH3 and linear MARCH3 mRNA were measured by RT-PCR at the indicated time points.

We identified circMARCH3 as a 449 bp exonic circRNA originating from back-splicing of exons 2 and 3 of the MARCH3 pre-mRNA via cloning and sequencing (**Figure 1E**; **Supplementary Figures S1A, B**). BLAST analysis revealed 95% nucleic acid sequence homology between human and mouse circMARCH3 (**Supplementary Figure S1C**). To verify that the amplified product was indeed a circRNA rather than a linear RNA derived from MARCH3, we designed divergent primers that cross the cyclization site and convergent primers that do not cross the site (**Figure 1F**). These primers were used to amplify cDNA and genomic DNA (gDNA) from A549 cells. A single clear band was amplified by the divergent primers in the cDNA sample but not in the gDNA, confirming that the circular structure of circMARCH3 (**Figure 1G**). Additionally, circMARCH3 was more resistant to RNase R than linear MARCH3 mRNA (**Figure 1H**). The actinomycin D treatment also demonstrated that the half-life of circMARCH3 was much longer than that of the linear MARCH3 transcript, confirming the high stability of circMARCH3 (**Figure 1I**). Together, these experiments demonstrate that circMARCH3 is a previously uncharacterized circRNA whose expression is induced following IAV infection, suggesting its potential role in IAV-host interaction.

### 3.2 CircMARCH3 expression is significantly upregulated by infections with several RNA and DNA viruses

Since circMARCH3 was induced by IAV PR8 infection *in vitro*, we next tested whether this induction also occurs *in vivo*. To this end, C57BL/6 mice were infected with IAV PR8, and peripheral blood and lung tissues were collected at four time points (0, 12, 24, and 48 h post-infection) to detect circMARCH3 expression. The results showed that circMARCH3 was continuously induced in the IAV PR8-infected mice (**Figures 2A, B**; **Supplementary Figures S2A, B**). To determine whether this induction is specific to the IAV PR8 strain, we further examined the response of circMARCH3 to other IAV subtypes. Infection of human A549 and 293T cells with the IAV WSN and CA/04 strains also led to a significant increase in circMARCH3 level (**Figure 2C**; **Supplementary Figures S2C, D**). We next investigated whether circMARCH3 induction depends on infection time and viral dose. A549 and 293T cells were infected with IAV CA/04, WSN and PR8 strains, and circMARCH3 expression was analyzed by RT-PCR and quantitative RT-PCR (qRT-PCR) at various time points and multiplicities of infection (MOI). The results showed that the expression of circMARCH3 in both cell lines was increased in time- and dose-dependent manners (**Figures 2D–I**; **Supplementary Figures S2E–H**). Moreover, murine NIH/3T3 cell line was used, and similar patterns of circMARCH3 expression were observed in the cells infected with IAV strain PR8 at different MOIs and different times (**Figures 2J, K**; **Supplementary Figures S2I, J**). Collectively, these results reveal that circMARCH3 is significantly upregulated by infections with different IAV strains of H1N1 across multiple human and murine cell lines.

**Figure 2:**
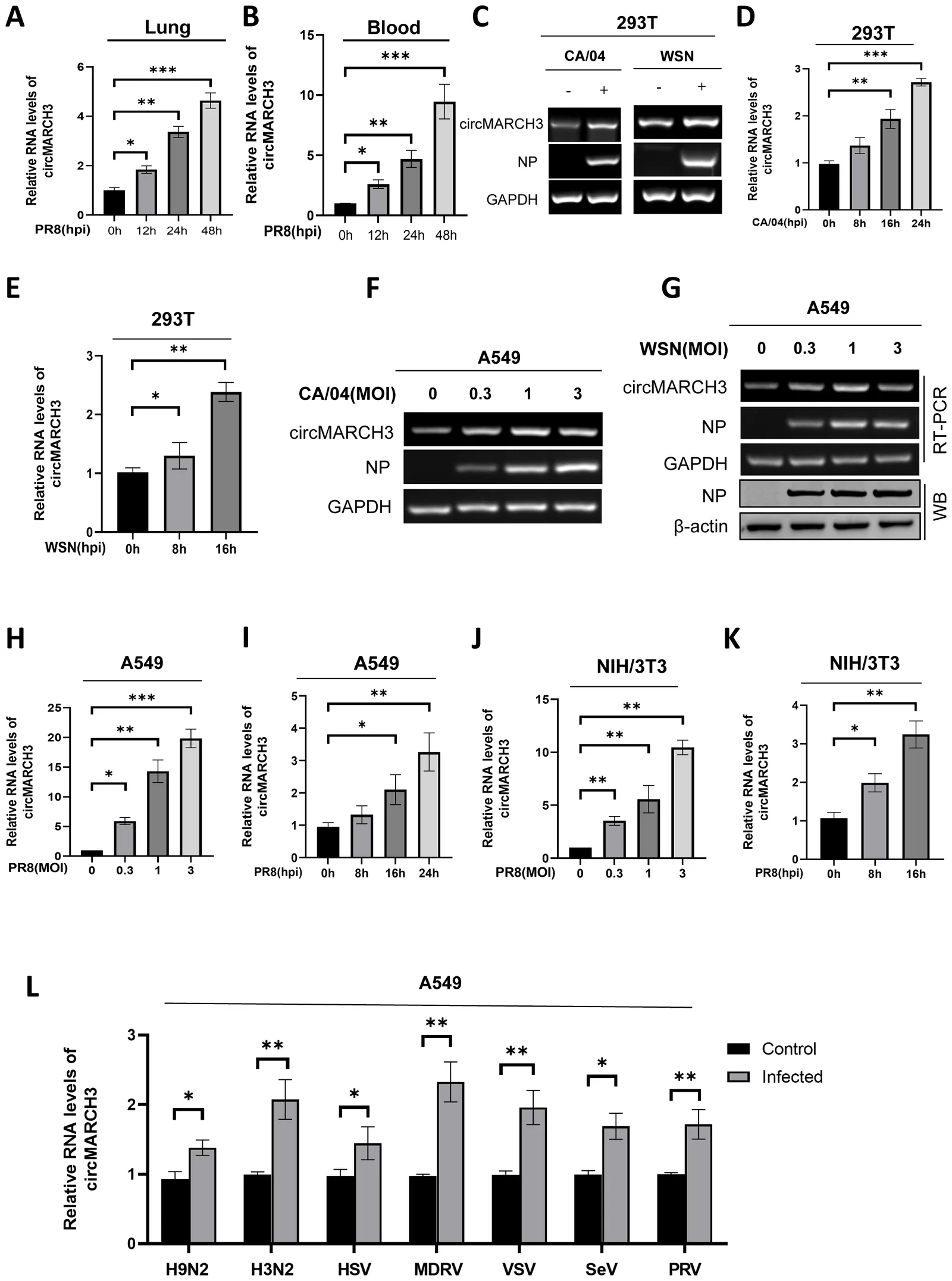
CircMARCH3 expression is upregulated by infections with a range of RNA and DNA viruses. **(A, B)** qRT-PCR detection of circMARCH3 expression in the lung tissues and peripheral blood of wild-type mice infected with IAV PR8 (MOI = 1) for 48 h. **(C)** 293T cell lines were infected with CA/04, WSN (MOI = 1) for 16 h. CircMARCH3 expression was assessed by RT-PCR. **(D, E)** 293T cell lines were infected with CA/04, WSN (MOI = 1), and samples were collected at the indicated time points. CircMARCH3 expression was assessed by qRT-PCR. **(F, G)** RT-PCR was performed to examine circMARCH3 expression in A549 cells infected with IAV CA/04 **(F)** or WSN **(G)** at indicated MOIs for 16 h. **(H, I)** qRT-PCR was performed to quantify circMARCH3 levels in PR8-infected A549 cells at indicated MOIs for 16 h **(H)** or at indicated time points **(I)**. **(J, K)** qRT-PCR was employed to detect the RNA levels of circMARCH3 in NIH/3T3 cells infected with PR8 at the indicated MOIs for 16 h **(J)** or at the indicated time points **(K)**. **(L)** A549 cell lines were infected with or without H9N2, H3N2, HSV, MDRV, VSV, SeV, or PRV (MOI = 1) for 16 h. qRT-PCR was employed to detect the RNA levels of circMARCH3. Data represent mean values ± SD (n = 3; *p < 0.05; **p < 0.01; ***p < 0.001).

We next determined whether circMARCH3 expression could be induced by other DNA or RNA viruses. To this end, A549 cells were infected with RNA viruses including Sendai virus (SeV), IAV subtypes H9N2 and H3N2, Vesicular stomatitis virus (VSV), Muscovy duck reovirus (MDRV), as well as DNA viruses including Herpes simplex virus (HSV) and Pseudorabies virus (PRV), followed by examining the expression of circMARCH3. Interestingly, infection with each of these viruses also upregulated the expression of circMARCH3 (**Figure 2L**). These findings indicate that circMARCH3 is a conserved circRNA, which can be broadly induced by diverse RNA and DNA viruses in various human and murine cells.

### 3.3 IAV-induced circMARCH3 expression is regulated by NF-κB signaling pathway

Since circMARCH3 expression is obviously induced within several hours post viral infection, we hypothesized that circMARCH3 expression may be regulated by innate immune signaling activated by the viral infection. To test this, we first transfected A549 cells with different concentrations of poly(I:C), a synthetic double-stranded RNA (dsRNA), and assessed the effect of poly(I:C) on circMARCH3 expression. We found that poly(I:C) significantly enhanced circMARCH3 expression, and that, as expected, it induced the production of ISG15 in a dose-dependent manner (**Figures 3A, B**). We then treated A549 cells with different concentrations of LPS, an activator of NF-κB pathway, and observed a dose-dependent increase in circMARCH3 and IL-6 expression (**Figures 3C, D**).

**Figure 3:**
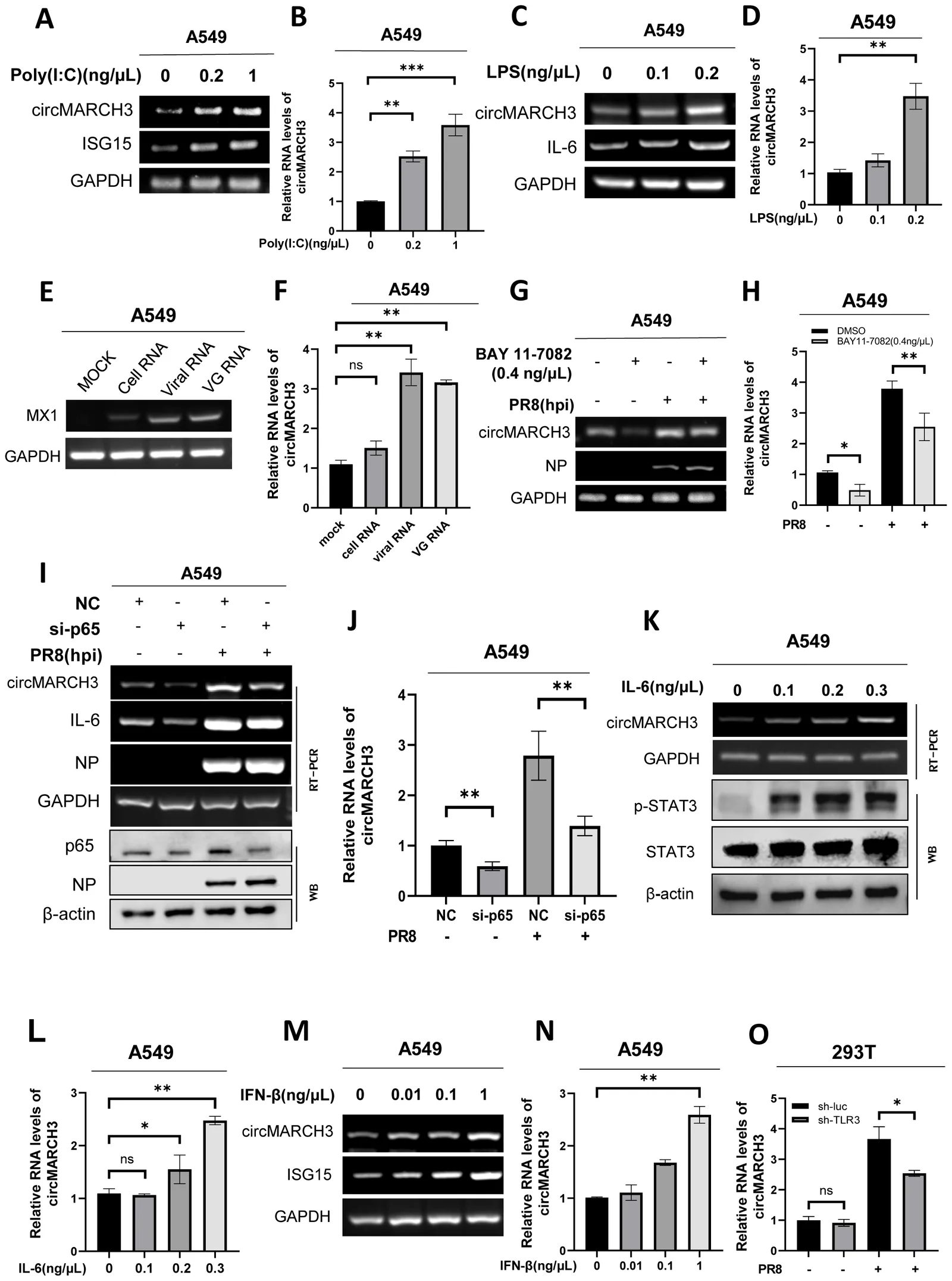
IAV-induced circMARCH3 expression is regulated by NF-κB signaling pathway. **(A, B)** A549 cells were transfected with poly (I: C) at indicated concentrations for 4 h. The levels of circMARCH3 were examined by RT-PCR **(A)** and qRT-PCR **(B)**. **(C, D)** A549 cells were treated with LPS at indicated concentrations for 8 h, and circMARCH3 expression was examined by RT-PCR **(C)** and qRT-PCR **(D)**. **(E, F)** Total RNA from either uninfected A549 cells (cell RNA), or PR8 infected-A549 (viral RNA), and isolated PR8 viral genomic RNA (VG-RNA) were extracted, respectively. The three RNA preparations were then transfected into A549 cells and incubated for 12 h. Levels of circMARCH3 were examined by RT-PCR **(E)** or qRT-PCR **(F)**. **(G, H)** A549 cells were pre-treated with BAY 11-7082 or DMSO (control) for 30 min before challenge with PR8 for 16 h. The RNA levels of circMARCH3 were determined by RT-PCR **(G)** or qRT-PCR **(H)**. **(I, J)** Control A549 cells and A549 cells with siRNA-mediated NF-kB p65 silencing were infected with PR8 for 16 h. Western blotting and RT-PCR were applied to verify the knockdown efficiency of NF-kB p65 **(I)**. The RNA levels of circMARCH3 were quantified by RT-PCR **(I)** and qRT-PCR **(J)**, respectively. **(K, L)** A549 cells were treated with IL-6 at indicated concentrations for 6 h, and the RNA levels of circMARCH3 were examined by RT-PCR **(K)** and qRT-PCR **(L)**. **(M, N)** Expression of circMARCH3 was examined by RT-PCR **(M)** and qRT-PCR **(N)** in A549 cells treated with IFN-β at indicated concentrations for 6 h. **(O)** 293T cells were transfected with shRNA plasmids targeting TLR3 (sh-TLR3) or luciferase (sh-luc, control) for 24 h, followed by IAV PR8 infection for 16 h. qRT-PCR was employed to quantify the RNA levels of circMARCH3. Data represent mean values ± SD (n = 3; ns, no significance; *p < 0.05; **p < 0.01; ***p < 0.001).

To further confirm that influenza virus RNA can upregulate circMARCH3 expression, we extracted total RNA from either uninfected A549 cells (Cell RNA), or PR8-infected A549 cells (Viral RNA), and isolated genomic RNA of influenza virus (VG-RNA), and transfected them into A549 cells, respectively. Strikingly, both Viral RNA and VG-RNA induced the expression of circMARCH3 as well as the production of MX1 in A549 cells (**Figures 3E, F**). To directly examine the role of NF-κB, we treated cells with NF-κB inhibitor BAY 11-7082 prior to IAV infection. Notably, BAY 11-7082 treatment significantly suppressed the virus-induced circMARCH3 expression (**Figures 3G, H**). Consistent with this, siRNA-mediated knockdown of the NF-κB subunit p65 also significantly diminished circMARCH3 expression (**Figures 3I, J**).

Given that NF-κB can regulate the expression of IL-6 and type I IFNs during IAV infection, we next asked whether IL-6 and IFN-β could upregulate circMARCH3 expression. Firstly, A549 cells were stimulated with various concentrations of IL-6, and a significant upregulation of circMARCH3 expression was observed in the cells (**Figures 3K, L**). Furthermore, we found that treating A549 cells with IFN-β also significantly increased circMARCH3 expression (**Figures 3M, N**). Because TLR3 is a key sensor for double-stranded RNA intermediates produced during IAV replication and can activate NF-κB-mediated immune response, we investigated if TLR3 was required for IAV-induced circMARCH3 expression. Indeed, knockdown of TLR3 caused a significant decrease in circMARCH3 level in 293T cells compared with the control following IAV infection (**Figure 3O**). Collectively, these results indicate that IAV-induced circMARCH3 expression is driven by the NF-κB-mediated innate immune signaling.

### 3.4 IAV-induced circMARCH3 expression facilitates viral replication

Next, we investigated the role of IAV-induced circMARCH3 in viral infection. For this, we examined whether modulation of circMARCH3 expression affects viral replication. Short hairpin RNAs (shRNAs) to specifically disrupt the expression of circMARCH3 in A549 and NIH/3T3 cells were designed, and transfection efficiency was verified by observing green fluorescent protein (GFP) expression via fluorescence microscopy (**Supplementary Figure S3A**). The results showed that knockdown of circMARCH3 had no effect on the expression of linear MARCH3 but significantly reduced viral NP mRNA and protein levels in IAV-infected A549 and NIH/3T3 cells compared to the control group (**Figures 4A, B**; **Supplementary Figure S3B**). Viral titers determined by the HA assay showed that circMARCH3 silencing resulted in a clearly lower viral load compared with the control (**Figures 4C, D**). This finding was further confirmed by PFA analysis (**Figures 4E, F**). These experiments demonstrated that silencing circMARCH3 impaired IAV replication.

**Figure 4:**
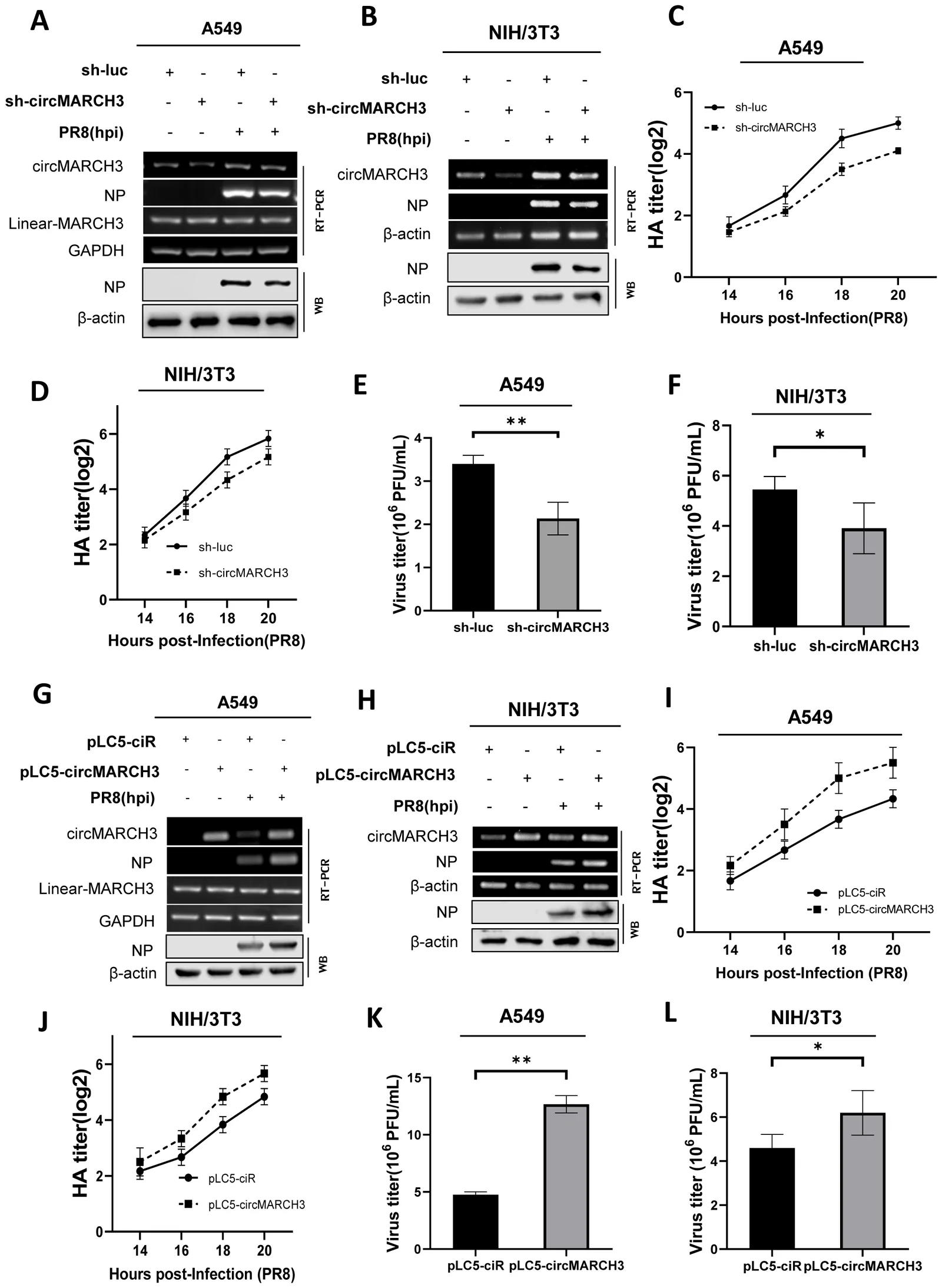
Modulation of circMARCH3 expression affects IAV replication. **(A–F)** A549 **(A, C, E)** or NIH/3T3 **(B, D, F)** cells were transfected with shRNA targeting circMARCH3 (sh-circMARCH3) or luciferase (sh-luc, control), and subsequently challenged with IAV PR8 (MOI = 1). RT-PCR was performed to verify circMARCH3 knockdown efficiency **(A, B)**. RT-PCR and Western blotting was utilized to assess IAV-NP levels **(A, B)**. Supernatants were collected at indicated time points. HA assay was performed to measure IAV titers in the supernatants of A549 **(C)** or NIH/3T3 **(D)** cells at indicated time points, and plaque-forming assay was employed to examine the IAV titers of supernatants from A549 **(E)** or NIH/3T3 **(F)** cells challenged with IAV PR8 for 18 h. **(G–L)** A549 **(G, I, K)** or NIH/3T3 **(H, J, L)** cells stably expressing control (pLC5-ciR) or circMARCH3 (pLC5-circMARCH3) were infected with IAV PR8 (MOI = 1). RT-PCR was utilized to assess the overexpression efficiency of circMARCH3 **(G, H)**. Supernatants from PR8-infected cells were collected at indicated time points. IAV titers in the supernatants of A549 **(I)** or NIH/3T3 **(J)** cells were measured at indicated time points by HA assay. IAV titers in the supernatants of PR8-infected circMARCH3-overexpressing A549 **(K)** or NIH/3T3 **(L)** cells at 18 h were measured by plaque-forming assay. Data represent mean values ± SD (n = 3; *p < 0.05; **p < 0.01).

Conversely, we used a pLC5-ciR overexpression system to determine whether forced expression of circMARCH3 could affect IAV replication. To this end, circMARCH3-overexpressing A549 and NIH/3T3 cell lines were generated (**Supplementary Figure S3C**), and the effects of circMARCH3 overexpression on IAV replication were evaluated. Compared with the control group, while overexpression of circMARCH3 had little effect on linear MARCH3 expression, the expression of IAV NP mRNA was upregulated in both circMARCH3-overexpressing A549 and NIH/3T3 cell (**Figures 4G, H**; **Supplementary Figure S3D**). Consistent with the transcriptional changes, Western blotting analysis further confirmed that NP protein levels were markedly elevated in the cells (**Figures 4G, H**). As shown in **Figures 4I, J**, the HA assay showed that circMARCH3-overexpressing cells had higher viral titers than control cells. Furthermore, the PFA analysis validated the observations (**Figures 4K, L**). These data indicate that IAV-induced circMARCH3 promotes viral replication in both human and mouse cells.

### 3.5 CircMARCH3 suppresses antiviral innate immunity by modulating NF-κB and type I IFN signaling

Above results reveal that circMARCH3 expression is rapidly increased upon activation of innate immune signaling and promotes IAV replication, we hypothesized that circMARCH3 might be involved in innate immune response to the viral infection. Since circMARCH3 expression was regulated by the NF-κB pathway, we first investigated whether it could regulate NF-κB-mediated antiviral innate immunity against IAV through a potential feedback mechanism. To test this hypothesis, we employed a dual-luciferase reporter assay to examine NF-κB and IFN-β promoter activity in circMARCH3-overexpressing 293T cells infected with IAV. Indeed, dual-luciferase reporter assays showed that both NF-κB and IFN-β promoter activities were significantly reduced in circMARCH3-overexpressing cells compared with control cells after IAV infection (**Figures 5A, B**). Furthermore, to define which specific component of the NF-κB/IFN pathway is targeted by circMARCH3, we co-transfected an NF-κB or IFN-β promoter with RIG-I or TBK1 overexpression plasmids into circMARCH3-overexpressing 293T cells. We found that circMARCH3 overexpression significantly suppressed NF-κB and IFN-β promoter activities induced by RIG-I or TBK1 (**Figures 5C, D**), suggesting that its targeting site may be located at the level of TBK1 or downstream of TBK1.

**Figure 5:**
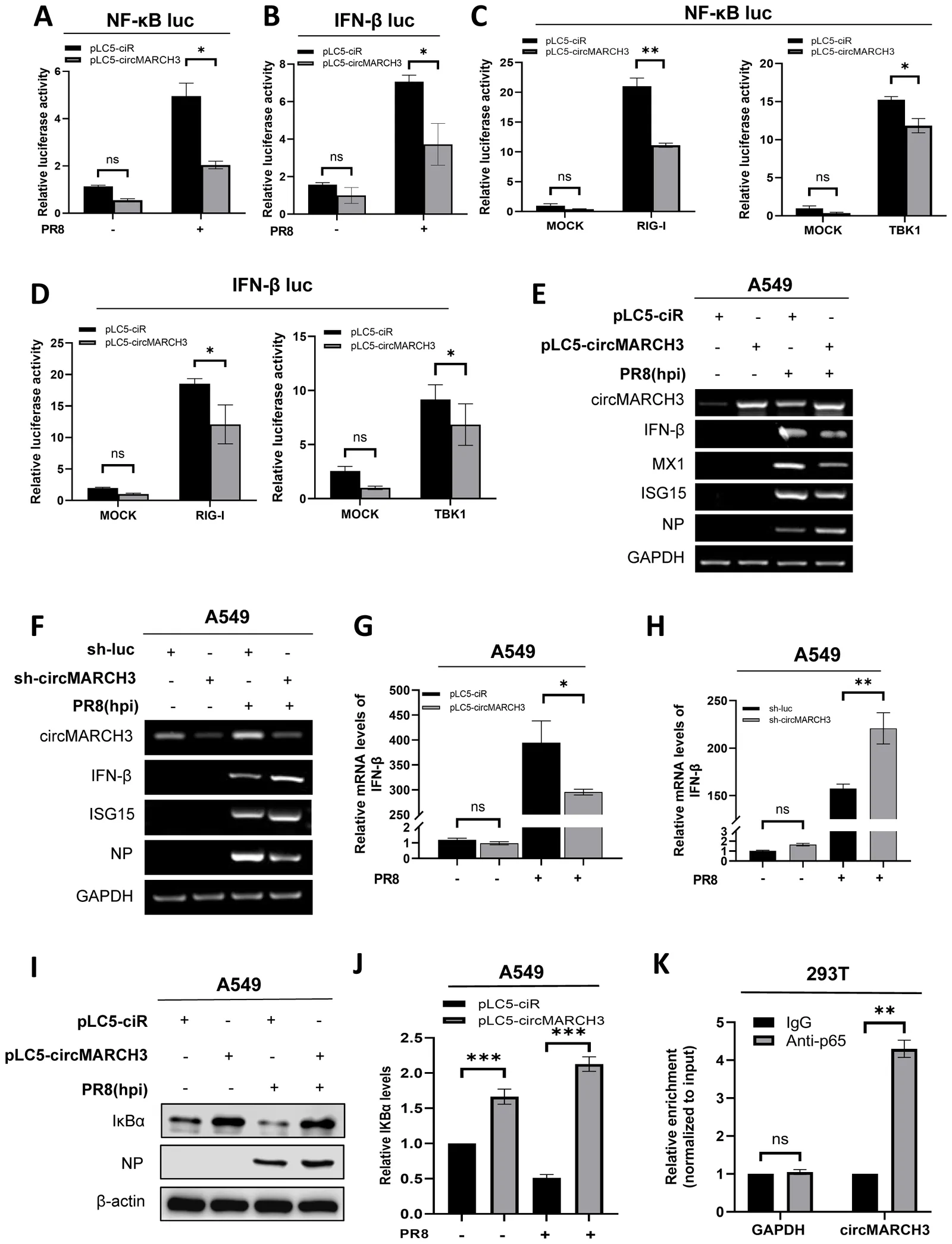
CircMARCH3 suppresses antiviral innate immunity involving NF-κB and type I IFN. **(A, B)** Control and circMARCH3-overexpressing 293T cells were transfected with NF-κB **(A)** or IFN-β **(B)** luciferase reporter along with pRL-TK plasmids. Following 24-h transfection, the cells were infected with or without PR8 (MOI = 1) for 16 h, followed by collection for luciferase assay. **(C, D)** 293T cells with control or circMARCH3-overexpresson were transfected with NF-κB **(C)** or IFN-β **(D)** luciferase reporter along with indicated plasmids. At 24 h post-transfection, the cells were harvested for luciferase assay. **(E, F)** RT-PCR was performed to detect the mRNA expression of IFN-β, MX1, and ISG15 in circMARCH3-overexpressing **(E)** or circMARCH3-silencing **(F)** A549 cells infected with or without IAV PR8 for 16 h. **(G, H)** The mRNA levels of IFN-β in circMARCH3-overexpressing **(G)** or circMARCH3-knockdowning **(H)** A549 cells infected with or without IAV PR8 were detected by qRT-PCR. **(I, J)** Western blotting was performed to detect IκBα protein levels in circMARCH3-overexpressing A549 cells infected with PR8 **(I)**. The protein levels of IκBα in **(I)** was quantitated by densitometry and normalized to β-actin levels **(J)**. **(K)** 293T cells were subjected to RIP using an anti-p65 antibody or control IgG, followed by qRT-PCR detection of circMARCH3. GAPDH mRNA was used as a negative control. Data represent mean values ± SD (n = 3; ns, no significance; *p < 0.05; **p < 0.01; ***p < 0.001).

Based on this finding, we next determined whether circMARCH3 affects the expression of downstream innate immune factors. The expression of IFN-β, MX1, and ISG15 was found to be decreased in circMARCH3-overexpressing cells compared with control cells after IAV infection (**Figure 5E**). Conversely, knockdown of circMARCH3 increased the expression of IFN-β and ISG15 (**Figure 5F**). These results were confirmed by qRT-PCR (**Figures 5G, H**). Since overexpression of circMARCH3 impaired the NF-κB signaling, we further investigated whether it affects the IκBα protein expression. The results showed that circMARCH3 overexpression elevated the protein levels of IκBα in the cells after IAV infection (**Figures 5I, J**). Furthermore, we performed an RNA immunoprecipitation (RIP) assay and observed that circMARCH3 was enriched by the anti-p65 antibody compared with normal IgG control (**Figure 5K**), suggesting an association between circMARCH3 and p65. Collectively, these data suggest that circMARCH3 promotes IAV replication by suppressing antiviral immune response involving the NF-κB and type I IFN signaling pathways.

### 3.6 CircMARCH3 functions as a miR-3657 sponge to upregulate ATG7 expression

Accumulating evidence has established that circRNAs can act as endogenous miRNA sponges, regulating the expression of downstream target genes through competitive adsorption of specific miRNAs. To determine whether circMARCH3 could act as a miRNA sponge, subcellular localization of circMARCH3 was analyzed. We performed nuclear-cytoplasmic fractionation in A549 cells and found that circMARCH3 was predominantly localized in the cytoplasm by RT-PCR and qRT-PCR (**Figures 6A, B**). To investigate the potential interactions between circMARCH3 and miRNAs, we performed miRNA-seq in circMARCH3-overexpressing and control cells. Sequencing data characterized the differential expression patterns of miRNAs, which were clustered based on log2-fold changes and visualized in a heatmap (**Figure 6C**). A total of 1086 miRNAs were identified, and 57 miRNAs exhibited significantly changed expression in circMARCH3-overexpressing cells (|log_2_FC|>1), including 29 downregulated miRNAs and 28 upregulated miRNAs. Among the differentially expressed miRNAs, miR-3657, a known negative regulator of ATG7 was significantly downregulated.

**Figure 6:**
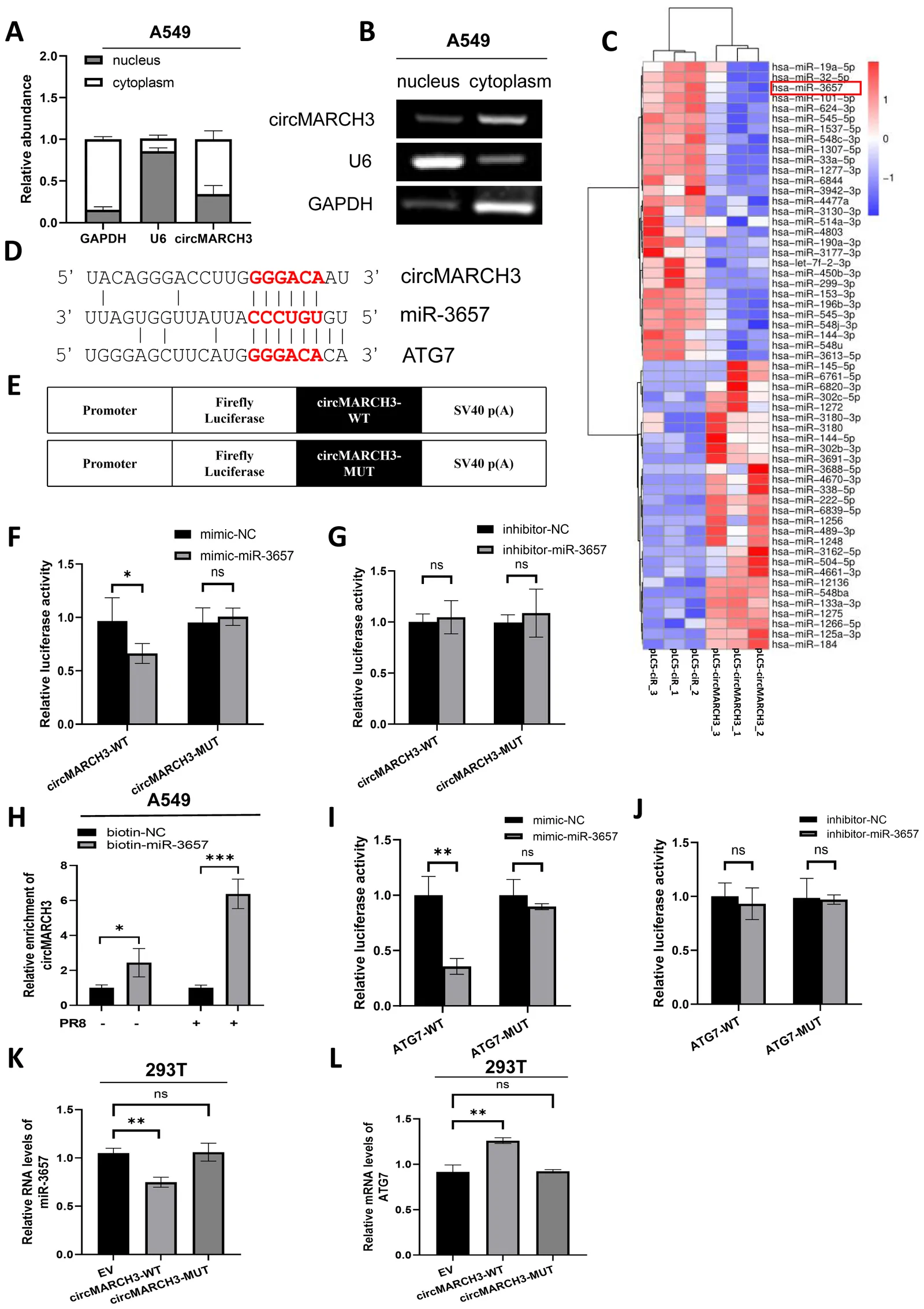
CircMARCH3 functions as a miR-3657 sponge to upregulate ATG7 expression. **(A, B)** Total RNA was isolated from the cytoplasmic and nuclear fractions of A549 cells using a nucleoplasmic separation kit. GAPDH and U6 were used as cytoplasmic and nuclear markers, respectively. The expression of circMARCH3 was verified by qRT-PCR **(A)** and RT-PCR **(B)**. **(C)** miRNA-seq analysis was conducted on control and circMARCH3-overexpressing 293T cells infected with PR8. Differentially expressed miRNAs are shown in the heatmap. **(D)** Sequences of circMARCH3 and ATG7 binding sites in miR-3657. **(E)** Wild-type circMARCH3 (circMARCH3-WT) or its mutants (circMARCH3-MUT) were cloned downstream of the Renilla luciferase gene in the vector pmiR-GLO. A schematic diagram shows the constructs generated for luciferase assays. **(F)** CircMARCH3-WT and circMARCH3-MUT cloned into pmiR-GLO vector were respectively transfected into 293T cells together with miR-3657 mimic (mimic-miR-3657) or negative control (mimic-NC), followed by the luciferase assay. **(G)** CircMARCH3-WT and circMARCH3-MUT in pmiR-GLO vector were transfected into 293T cells together with miR-3657 inhibitor (inhibitor-miR-3657) or negative control (inhibitor-NC), followed by the luciferase assay. **(H)** A549 cells were transfected with biotinylated miR-3657 mimics (biotin-miR-3657) or a negative control (biotin-NC). After 48 h, the cells were infected with IAV PR8 for 24 h, followed by a biotin-based miRNA pull-down assay, and the enrichment of circMARCH3 was determined by qRT-PCR. **(I)** Wild-type ATG7 (ATG7-WT) or a mutant with altered miR-3657 response elements (ATG7-MUT) was cloned into the pmiR-GLO vector and co-transfected into 293T cells with mimic-miR-3657 or mimic-NC. Relative luciferase activity was determined. **(J)** 293T cells were co-transfected with the pmiR-GLO vector containing ATG7-WT or ATG7-MUT, together with inhibitor-miR-3657 or inhibitor-NC. Relative luciferase activity was determined. **(K, L)** The expression levels of miR-3657 **(K)** and ATG7 **(L)** were evaluated by qRT-PCR in 293T cells stably expressing circMARCH3-WT, circMARCH3-MUT, or control vector. Data represent mean values ± SD (n = 3; ns, no significance; *p < 0.05; **p < 0.01).

Next, potential binding sites were predicted between circMARCH3 and miR-3657, as well as between miR-3657 and ATG7 by using the miRanda database (**Figure 6D**). To validate whether circMARCH3 served as a sponge for miR-3657, wild-type circMARCH3 (circMARCH3-WT) or its mutants with altered miRNA-binding sites (circMARCH3-MUT) were cloned into the 3’ UTR of the Renilla luciferase gene in the pmiR-GLO vector, respectively (**Figure 6E**). These plasmids were then co-transfected with corresponding miRNA mimics or negative controls into 293T cells, and four experimental groups were set up: circMARCH3-WT + mimic-NC, circMARCH3-WT + mimic-miR-3657, circMARCH3-MUT + mimic-NC, and circMARCH3-MUT + mimic-miR-3657. Dual-luciferase assays revealed that co-transfection of the wild-type reporter with the miR-3657 mimic significantly reduced relative luciferase activity compared with the control group, whereas mutation of the predicted binding site abolished this inhibitory effect (**Figure 6F**). Parallel experiments conducted with a miR-3657 inhibitor (inhibitor-miR-3657) showed no appreciable differences in luciferase expression among any of the experimental groups (**Figure 6G**). To further confirm the interaction between circMARCH3 and miR-3657, we performed a biotin-labeled miRNA pull-down assay. The results showed that circMARCH3 was efficiently enriched in pull-down fraction by the biotinylated miR-3657 mimics compared with the control (**Figure 6H**), indicating a direct interaction between circMARCH3 and miR-3657. Collectively, these results confirmed that circMARCH3 acted as a molecular sponge for miR-3657 and that the interaction occurred between the predicted binding sites.

An identical experimental strategy was then employed to examine the interaction between ATG7 and miR-3657. For this, wild-type ATG7 (ATG7-WT) and a binding site-deficient mutant (ATG7-MUT) were inserted into the pmiR-GLO vector and co-transfected into 293T cells with the corresponding miRNA mimics or inhibitor. Consistent with the results obtained for circMARCH3, co-transfection of ATG7-WT with the miR-3657 mimic led to a marked decrease in luciferase activity, whereas this inhibitory effect was completely abrogated upon mutation of the putative miR-3657 binding sites (**Figure 6I**). Moreover, the miR-3657 inhibitor failed to alter reporter expression across all groups (**Figure 6J**). Besides, we constructed circMARCH3-WT and circMARCH3-MUT plasmids in the pLC5-ciR vector and examined their effects on ATG7 and miR-3657 expression in 293T cells. As expected, circMARCH3-WT had significant effects on the ATG7 and miR-3657 expression (**Figures 6K, L**). However, levels of ATG7 and miR-3657 in circMARCH3-MUT cells were comparable to the control cells (**Figures 6K, L**). Taken together, these findings demonstrate that ATG7 is a direct target of miR-3657 and that circMARCH3 acts as a ceRNA by sponging miR-3657, thereby relieving miR-3657-mediated repression of ATG7 expression.

### 3.7 The circMARCH3/miR-3657/ATG7 axis regulates IAV replication

To investigate the regulatory role of the circMARCH3/miR-3657/ATG7 axis in IAV replication, circMARCH3-overexpressing cells and control cells were infected with the IAV PR8 strain. The expression levels of miR-3657 and ATG7 were subsequently assessed. We observed that circMARCH3 overexpression led to a significant increase in ATG7 mRNA and protein levels. Notably, circMARCH3-mediated upregulation of ATG7 promoted the conversion of LC3-I to LC3-II and caused an increase in p62 degradation, indicating that autophagy is enhanced by the circMARCH3 (**Figure 7A**). As expected, circMARCH3 overexpression caused an increase in IAV NP protein. Conversely, circMARCH3 knockdown resulted in the opposite effects, characterized by downregulated ATG7 expression, reduced LC3-II accumulation, and decreased NP protein (**Figure 7B**). Consistent with above finding, circMARCH3 overexpression led to a significant decrease in miR-3657 expression, whereas its knockdown upregulated miR-3657 levels (**Figures 7C, D**).

**Figure 7:**
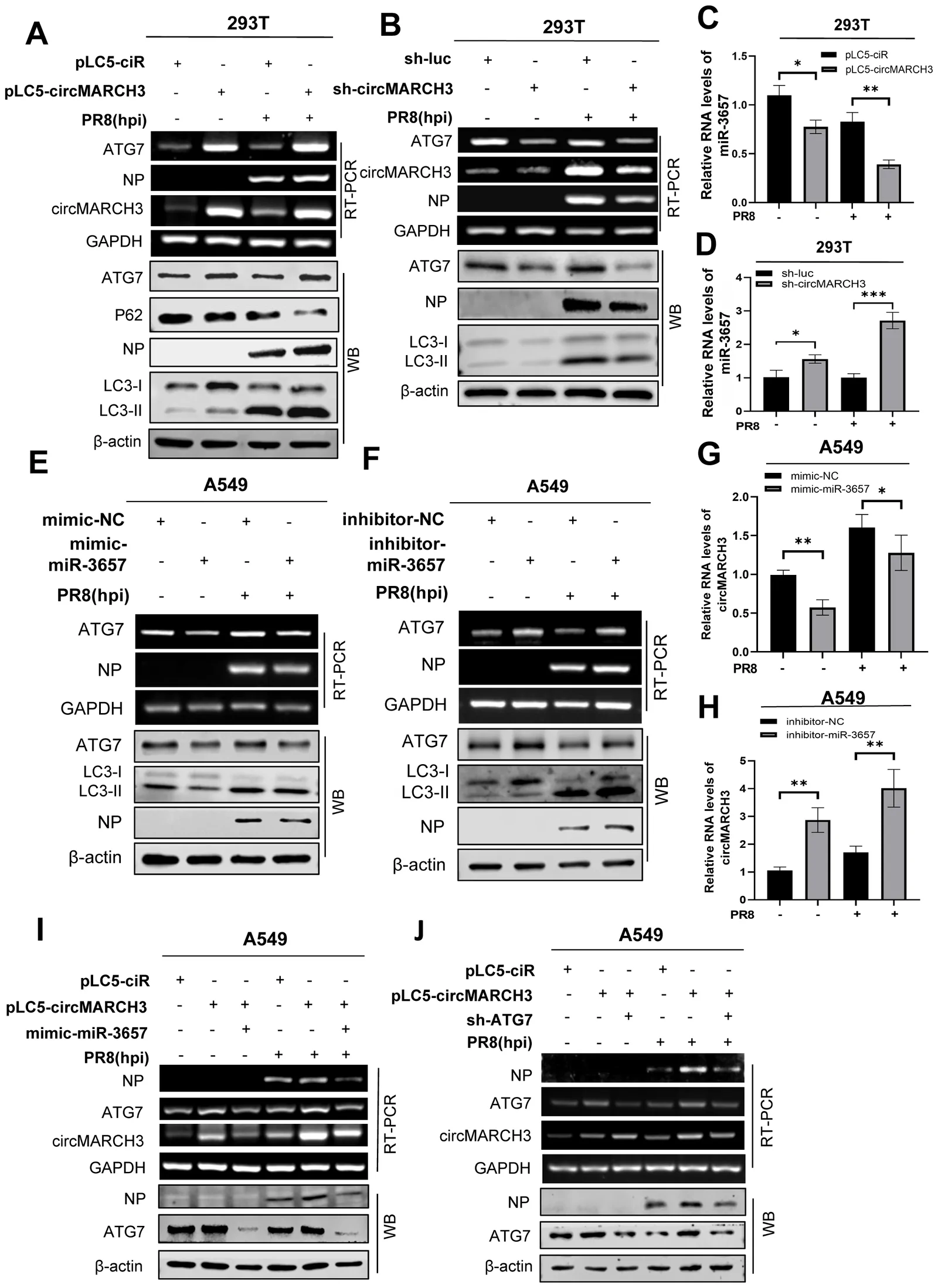
The circMARCH3/miR-3657/ATG7 axis regulates IAV replication. **(A, B)** RT-PCR and Western blotting were performed to determine the mRNA and protein levels of NP, ATG7 and P62 respectively, in circMARCH3-overexpressing **(A)**, or circMARCH3-silencing **(B)** 293T cells infected with PR8 (MOI = 1) for 16 h. **(C, D)** qRT-PCR was used to detect miR-3657 expression in circMARCH3-overexpressing **(C)** or circMARCH3-knockdown **(D)** 293T cells. **(E)** A549 cells transfected with mimic-miR-3657 or mimic-NC (50 μM) for 24 h, followed by PR8 infection for 16 h. ATG7 expression was examined by RT-PCR and Western blotting. **(F)** A549 cells were transfected with inhibitor-miR-3657 or inhibitor-NC (50 μM) for 24 h, followed by infection with PR8 for 16 h, and ATG7 expression was detected by RT-PCR and Western blotting. **(G)** qRT-PCR was performed to measure the expression of circMARCH3 in A549 cells transfected with mimic-3657 or mimic-NC (50 μM) for 24 h, followed by PR8 infection for 16 h. **(H)** A549 cells were transfected with inhibitor-miR-3657 or inhibitor-NC (50 μM) for 24 h, followed by IAV PR8 infection for 16 h. The RNA levels of circMARCH3 were measured by qRT-PCR. **(I)** RT-PCR and Western blotting were used to detect the mRNA and protein levels of NP and ATG7 in A549 cells co-transfected with pLC5-circMARCH3 and miR-3657 mimics, followed by PR8 infection. **(J)** RT-PCR and Western blotting were used to detect the mRNA and protein levels of NP and ATG7 in A549 cells co-transfected with pLC5-circMARCH3 and sh-ATG7, followed by PR8 infection. Data represent mean values ± SD (n = 3; *p < 0.05; **p < 0.01).

To further substantiate the regulatory interplay within the circMARCH3/miR-3657/ATG7 axis and its impact on IAV replication, A549 cells were transfected with a miR-3657 mimic or a specific miR-3657 inhibitor and detected the expression of ATG7 and the viral NP. Indeed, transfection of miR-3657 mimic clearly downregulated the abundance of ATG7 and concurrently suppressed IAV NP expression (**Figure 7E**; **Supplementary Figure S4A**). In contrast, inhibition of miR-3657 resulted in upregulation of ATG7 and NP levels (**Figure 7F**; **Supplementary Figure S4B**). Similarly, transfection of miR-3657 mimic reduced the expression of endogenous circMARCH3, but treatment with miR-3657 inhibitor increased the circMARCH3 levels (**Figures 7G, H**). To confirm these observations, 293T cells were employed and similar results were obtained from the experiments using the 293T cell model (**Supplementary Figures S4C, D**).

Furthermore, we directly addressed the relationship between miR-3657, ATG7, and circMARCH3. In circMARCH3-overexpressing cells, transfection with a miR-3657 mimic attenuated the promoting effect of circMARCH3 on IAV replication (**Figure 7I**). Similarly, knockdown of ATG7 in circMARCH3-overexpressing cells also reduced the effect of circMARCH3 on IAV replication (**Figure 7J**; **Supplementary Figure S4E**). Collectively, these results indicate that the circMARCH3/miR-3657/ATG7 axis plays an important role in regulating IAV replication.

## 4 Discussion

CircRNAs are a recently discovered class of endogenous non-coding RNA molecules characterized by a covalently closed loop structure. Accumulating evidence indicates that thousands of circRNAs are differentially expressed during viral infection and play key roles in modulating host-virus interactions and viral pathogenesis (49, 50). In particular, host-derived circRNAs are increasingly recognized as key regulators of influenza virus infection. Some circRNAs have been shown to regulate innate immunity or autophagy during the IAV infection. For example, circRNA-MerTK inhibits the production of IFN-β, thereby promoting IAV proliferation (45). IAV infection can induce autophagy in host cells, and circ-GATAD2 regulates IAV H1N1 replication through vacuolar protein sorting 34 (VPS34)-dependent autophagy (51). Numerous recent studies have revealed that circRNAs are implicated in the ceRNA regulatory network by acting as miRNA sponges, thereby affecting multiple biological processes such as immunity against viral infection. For instance, circRNA-0050463 enhances the expression of EEF1A1 by sponging miR-33b-5p, thereby promoting IAV replication; CircRNA AIVR sponges miR-330-3p to regulate the IAV replication by modulating IFN-β production (44, 52). Despite its importance, functional involvement of circRNAs in IAV infection remains largely unexplored. Moreover, ceRNA networks are critical for various cellular processes, but limited information is available about the precise mechanisms by which circRNA-involved ceRNA networks modulate IAV pathogenesis.

In this study, we identified a novel circRNA, circMARCH3, whose expression was markedly induced upon IAV and several other viral infections. We established a critical role for circMARCH3 in regulating host innate immunity during IAV infection. CircMARCH3 modulated IAV replication likely through two distinct but potentially cooperative mechanisms: a negative feedback loop suppressing NF-κB-mediated immune responses, and a competitive endogenous RNA axis that cross-regulates ATG7 expression via miR-3657/ATG7 pathway. Our studies demonstrated that circMARCH3 can stabilize ATG7 mRNA by sponging miR-3657, suggesting that circMARCH3 is involved in regulating ATG7-mediated autophagy, thereby enhancing IAV replication.

In this study, we observed that circMARCH3 upregulation was dependent on the NF-κB signaling pathway. Specifically, pharmacological inhibition of NF-κB (BAY 11-7082) and siRNA-mediated knockdown of p65 both significantly diminished virus-induced circMARCH3 expression, indicating that NF-κB activation is required for circMARCH3 induction. Interestingly, virus-induced expression of circMARCH3 in turn inhibited NF-κB activity and downstream IFN-β production, suggesting a negative feedback loop. NF-κB is a critical transcription factor driving pro-inflammatory and antiviral gene expression, and its activity is strictly controlled in response to pathogenic infection to prevent excessive inflammation and tissue damage. Therefore, the NF-κB/circMARCH3 negative feedback loop may be a host self-protective mechanism during the immune responses induced by IAV infection. However, viruses often exploit such loops to dampen host defenses. For example, several viral proteins or virus-induced host factors have been reported to interfere with NF-κB or IRF3/7 signaling (53–55). For circMARCH3, virus-induced, NF-κB-dependent expression may serve as an intrinsic negative regulator of the NF-κB pathway, thereby creating a self-protective mechanism in antiviral immunity. On the other hand, IAV may hijack the pathway to establish a state for the virus to replicate more efficiently. The finding that NF-κB induces circMARCH3 expression, while circMARCH3 in turn suppresses NF-κB signaling during IAV infection, suggests the existence of a complex regulatory interplay between the virus and the host.

However, the precise mechanism by which NF-κB induces circMARCH3 remains to be determined. Our data do not distinguish whether NF-κB directly regulates MARCH3 transcription, modulates spliceosomal components, or specifically enhances back-splicing. Further experiments, including ChIP-qPCR for p65, promoter luciferase assays, and assessment of RNA-binding proteins involved in circRNA biogenesis, will be performed in future studies to clarify this point.

Of note, circMARCH3 expression was also significantly induced by infections with several other viruses including both DNA viruses (e.g., HSV and PRV) and RNA viruses (e.g., MDRV and SeV), suggesting that circMARCH3 might have broad-spectrum effects on viral infection and pathogenesis. This pan-viral induction raises the question of whether it reflects a conserved host stress response or a co-evolved immune evasion strategy shared by multiple viruses. Given that circRNA biogenesis is regulated by innate immune and cellular stress pathways, and that many viruses modulate host splicing and RNA processing machinery, both possibilities remain plausible. Distinguishing between them will require further analyses across virus families and host species, as well as assessment of the evolutionary conservation of the regulatory elements controlling circMARCH3 biogenesis. In this study, we focused on elucidating the role of circMARCH3 in IAV replication. Whether circMARCH3 exerts similar regulatory effects during infection with other viruses remains to be determined and warrants further investigation.

It should be noted that our mechanistic studies focused primarily on the IAV PR8 strain. Whether circMARCH3 exerts conserved functions across seasonal H1N1, H3N2, highly pathogenic avian influenza viruses, or influenza B virus remains to be determined. Comparative analyses across influenza subtypes are required to clarify whether circMARCH3 represents a universal or strain-specific host factor. In addition, our results were obtained mainly from immortalized cell lines and a mouse infection model. Validation in primary human airway epithelial cells, differentiated air–liquid interface cultures, ex vivo respiratory tissues, and clinical samples from influenza patients is essential to establish the physiological and translational relevance of circMARCH3, which is worth further investigations.

Our transcriptome analysis revealed that 57 miRNAs exhibited significantly changed expression in circMARCH3-overexpressing cells, including 29 downregulated and 28 upregulated miRNAs. Functional assays confirmed that circMARCH3 upregulated ATG7 transcription by sponging miR-3657, thereby promoting autophagy, which is consistent with previous reported by Ma et al. (56). ATG7 is an essential effector enzyme for autophagosome vesicle elongation and plays a vital role in regulating viral infection and pathogenesis (57). Notably, autophagy exerts a dual role in viral infection: moderate autophagy facilitates the clearance of intracellular viruses (58, 59), whereas certain viruses, including IAV, have evolved strategies to hijack the autophagic machinery to enhance their own replication or release (60–62). Our studies provide further evidence that circMARCH3-mediated ATG7 upregulation correlated with increased viral production. Importantly, the circMARCH3/miR-3657/ATG7 axis can operate independently of the NF-κB pathway. This independence suggests that circMARCH3 promotes IAV replication through at least two parallel routes: dampening IFN-β production via NF-κB inhibition and enhancing autophagic flux via ATG7 upregulation. Such dual effects can explain why circMARCH3 has a relatively strong virus-promoting effect. It is worth noting that expression of total 57 miRNAs are significantly changed in circMARCH3-overexpressing cells. However, the interactions between circMARCH3 and other miRNAs remain unclear and require further investigation, and the functional relevance of these miRNAs in circMARCH3-mediated regulation of viral infection is worthy of exploration.

In summary, this study provides new insights into the roles of circRNA in IAV infection. We identified circMARCH3 as a virus-inducible circRNA that facilitates IAV replication through two distinct mechanisms: suppression of antiviral innate immunity via NF-κB-mediated negative feedback loop and promotion of IAV-induced autophagy through circMARCH3/miR-3657/ATG7 axis. These findings expand our understanding of the molecular mechanisms by which circRNAs contribute to influenza virus pathogenesis. Moreover, the identification of virus-induced circRNAs and characterization of the ceRNA networks in which they participate may facilitate the discovery of novel therapeutic targets for antiviral intervention.

## Data Availability

The datasets presented in this study can be found in online repositories. The names of the repository/repositories and accession number(s) can be found below: https://www.ncbi.nlm.nih.gov/geo/, GSE210688 https://www.ncbi.nlm.nih.gov/sra/, PRJNA1456839.
