# Supplementary material for: Circular RNA MARCH3 promotes influenza A virus replication by coordinating NF-κB negative feedback and the miR-3657/ATG7 axis
Source: Front Immunol. 2026 Oct 22;17:1948323. doi: 10.3389/fimmu.2026.1948323 (PMC13736778; doi:10.3389/fimmu.2026.1948323)
Supplement: Supplementary file 1 [file DataSheet1.pdf]

Supplementary Material

1 Supplementary Figures

Figure S1 Structural and sequence conservation of circMARCH3 in human and mouse. (A, B) The schematic drawing represents the human homolog (A), and the mouse homolog (B) of circMARCH3 formed by backsplicing between exon 2 and exon 3 of MARCH3 pre-mRNA. Schematic representation was generated by circPrimer2.0. (C) A multiple sequence alignment of human circMARCH3 (hsa-circMARCH3) and the mouse circMARCH3 homolog (mmu-circMARCH3), was made using basic local alignment search tool (BLAST, https://blast.ncbi.nlm.nih.gov/Blast.cgi).

Figure S2 Induction of circMARCH3 expression by infections with different IAVs. (A, B) RT-PCR detection of circMARCH3 expression in the lung tissues (A) and peripheral blood (B) of wild-type mice infected with IAV PR8 (MOI = 1) for 48 h. (C, D) qRT-PCR was employed to detect the RNA levels of circMARCH3 in A549 cells infected with CA/04 (C) or WSN (D). (E, F) RT-PCR was used to determine the RNA levels of circMARCH3 and IAV-NP in the A549 cell lines challenged with IAV CA/04 (E) or WSN (F) at the indicated time points. (G, H) RT-PCR was used to determine the RNA levels of circMARCH3 and IAV-NP in A549 cells challenged with IAV PR8 at indicated MOIs for 16 h (G) or at indicated time points (H). (I, J) Expression of circMARCH3 was examined by RT-PCR in NIH/3T3 cells infected with IAV PR8 at indicated MOIs for 16 h (I) or for indicated time points (J). Data represent mean values ± SD (n = 3; *, $p < 0.05$; **, $p < 0.01$).

Figure S3 Functional analysis of circMARCH3 in regulating IAV replication. (A) The transduction efficiency of A549 and NIH/3T3 cells stably expressing sh-circMARCH3 or luciferase control (sh-luc), was measured by visualizing GFP fluorescence activity. (B) The mRNA levels of NP

in circMARCH3-knockdown NIH/3T3 cells were detected by qRT-PCR. (C) The transduction efficiency of A549 and NIH/3T3 cells stably expressing circMARCH3 via the pLC5-ciR vector (pLC5-circMARCH3) and of the corresponding control cells (pLC5-ciR). (D) The mRNA levels of NP in circMARCH3-overexpressing NIH/3T3 cells were detected by qRT-PCR. Data represent mean values ± SD (n = 3; *, $p < 0.05$).

Figure S4 Regulation of IAV replication by the circMARCH3/miR-3657/ATG7 axis. (A) A549 cells transfected with miR-3657 mimic (mimic-miR-3657) or negative control (mimic-NC) at 50 μM for 24 h, followed by IAV PR8 infection for 16 h. The mRNA levels of NP were measured by qRT-PCR. (B) A549 cells transfected with miR-3657 inhibitor (inhibitor-miR-3657) or negative control (inhibitor-NC) at 50 μM for 24 h, followed by IAV PR8 infection for 16 h. The expression level of NP was measured by qRT-PCR. (C) 293T cells were transfected with mimic-miR-3657 or mimic-NC (50 μM) for 24 h and then infected with IAV PR8 for 16 h. ATG7 expression was detected by RT-PCR and western blotting, and circMARCH3 expression was detected by RT-PCR. (D) 293T cells were transfected with inhibitor-miR-3657 or inhibitor-NC (50 μM) for 24 h and then infected with IAV PR8 for 16 h. CircMARCH3 expression was examined by RT-PCR, and ATG7 expression was detected by RT-PCR and western blotting. (E) IAV titers in the supernatants of PR8-infected circMARCH3-overexpressing A549 cells with or without ATG7 knockdown were measured at the indicated time points by HA assay (MOI = 1). Data represent mean values ± SD (n = 3; *, $p < 0.05$; **, $p < 0.01$).

Figure S1

A

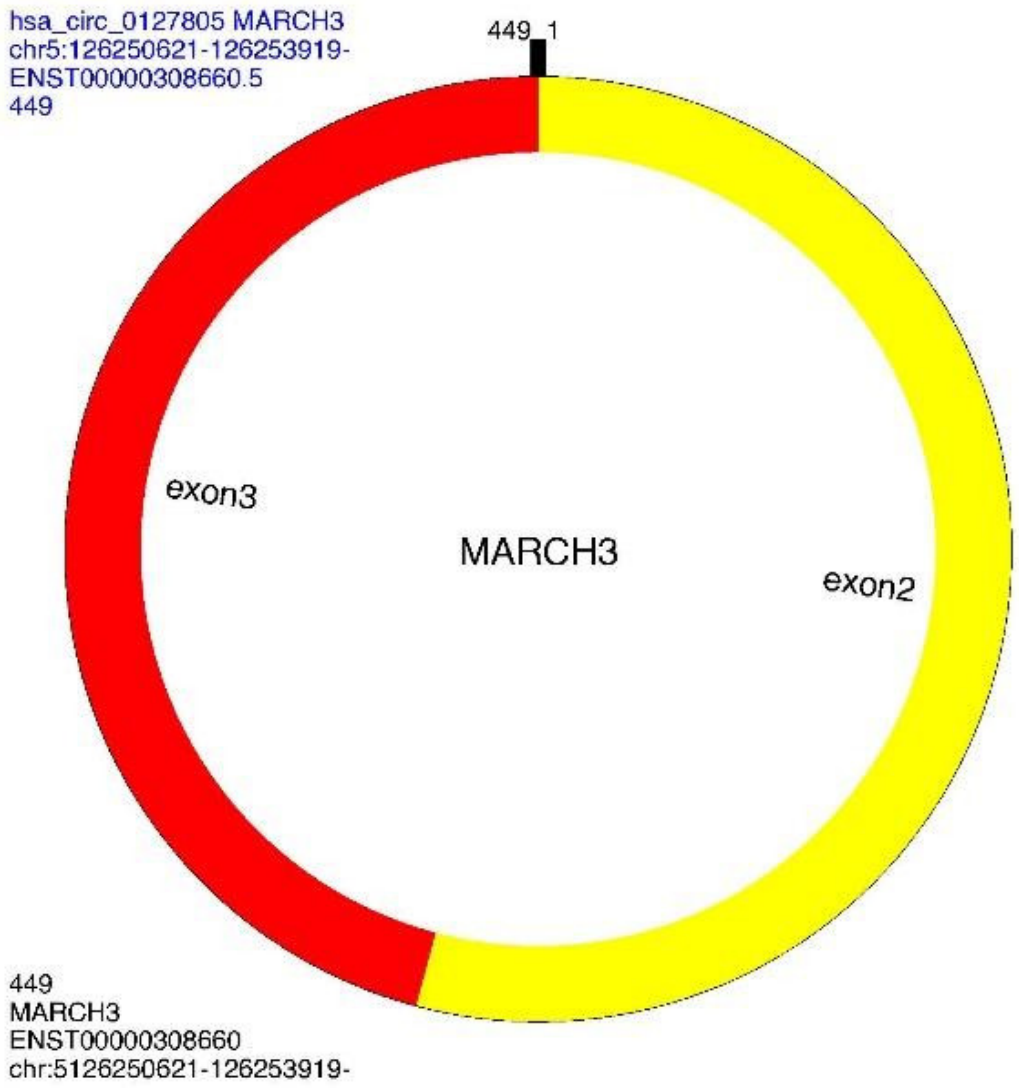

B

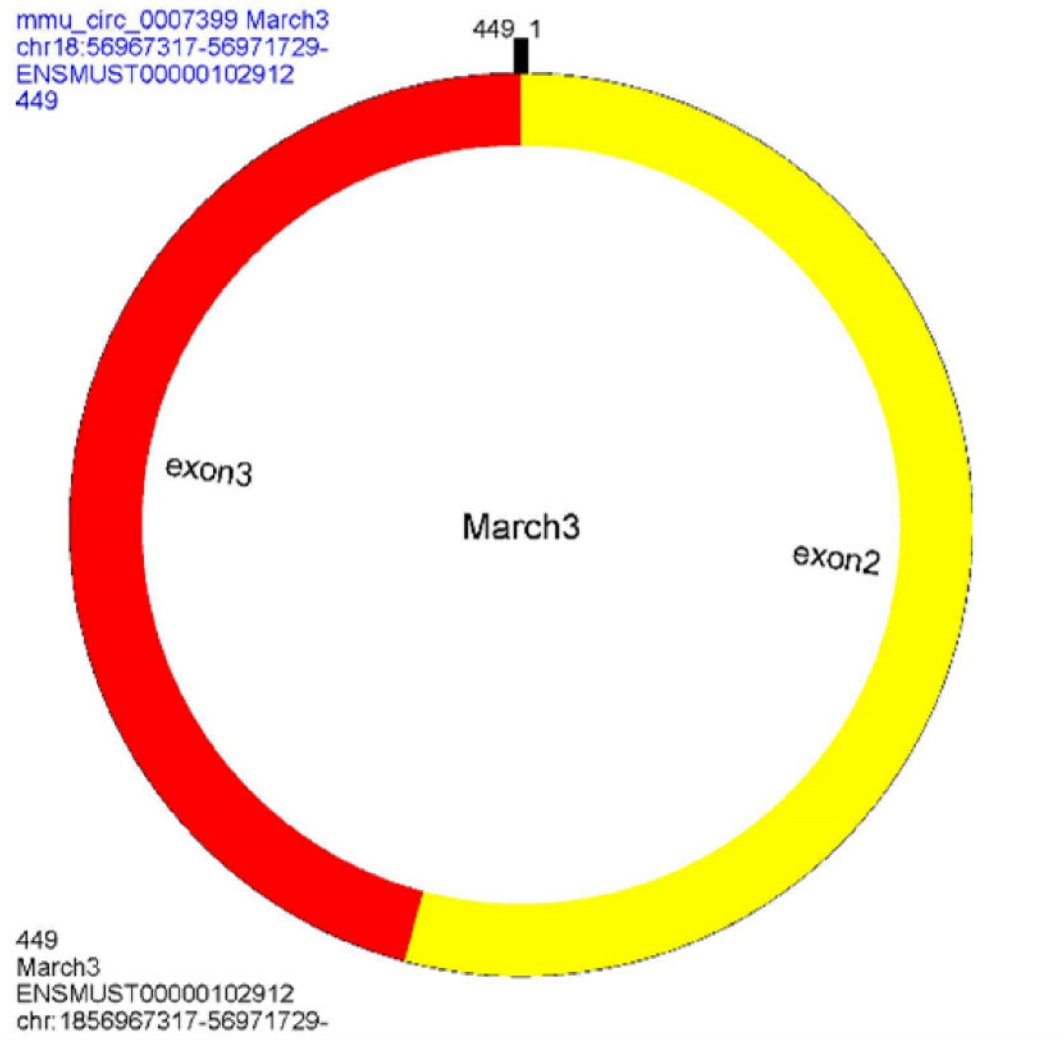

C

Score	Expect	Identities	Gaps	Strand
708 bits(383)	0.0	427/449(95%)	0/449(0%)	Plus/Plus

```
Hsa-circMARCH3   1    ATGGAAATCCTGTATGAATGTGGAAGCCATTAGCAGAGTAATTGCTGTCTGTTACCATGA  60
Mmu-circMARCH3   1    ATGGACGTCCTCTATGAATGTGGAAGCCATTAACCGAGTAATTGCTGTCTGTTGCCATGA  60

Hsa-circMARCH3   61   CAACCAGCCGCTGCAGTCACCTGCCCGAAGTCCTGCCAGACTGCACCAGCTCAGCTGCAC  120
Mmu-circMARCH3   61   CAACCAGTCGCTGCAGTCACCTGCCCGAAGTCTTGCCAGACTGCACCAGCTCAGCCGCGC  120

Hsa-circMARCH3   121  CCGTGGTGAAGACGGTGGAGGATTGTGGCAGCCTAGTGAATGGGCAGCCGCAGTATGTCA  180
Mmu-circMARCH3   121  CTGTGGTGAAGACGGTGGAGGATTGTGGCAGCCTAGTGAATGGGCAGCCACAGTATGTCA  180

Hsa-circMARCH3   181  TGCAAGTTTCAGCCAAGGACGGGCAGCTGCTGTCAACAGTAGTGCGGACTCTTGCCACCC  240
Mmu-circMARCH3   181  TGCAAGTTTCAGCCAAGGACGGGCAGCTGCTGTCAACAGTAGTGCGGACTCTTGCCACCC  240

Hsa-circMARCH3   241  AGAGCCCCTTCAATGACCGGCCGATGTGCAGGATCTGCCACGAGGGCAGCAGCCAAGAGG  300
Mmu-circMARCH3   241  AGAGCCCCTTCAATGACCGGCCAATGTGCAGGATCTGCCATGAGGGAAGCAGCCAAGAGG  300

Hsa-circMARCH3   301  ACTTGCTCTCTCCATGTGAATGTACAGGGACCTTGGGGACAATTCATCGGAGCTGCCTGG  360
Mmu-circMARCH3   301  ACTTACTCTCTCCGTGTGAATGTACTGGGACCTTGGGGACCATCCATCGGAGCTGCCTGG  360

Hsa-circMARCH3   361  AGCACTGGCTGTCATCCTCAAACACCAGCTACTGTGAACTCTGCCACTTCAGGTTTGCAG  420
Mmu-circMARCH3   361  AGCACTGGCTGTCATCCTCAAATACCAGCTACTGTGAACTCTGCCACTTCAGGTTTGCAG  420

Hsa-circMARCH3   421  TCGAGCGCAAACCCAGGCCGTTAGTGGAG  449
Mmu-circMARCH3   421  TGGAGCGCAAACCCAGGCCGTTAGTGGAG  449
```

Figure S2

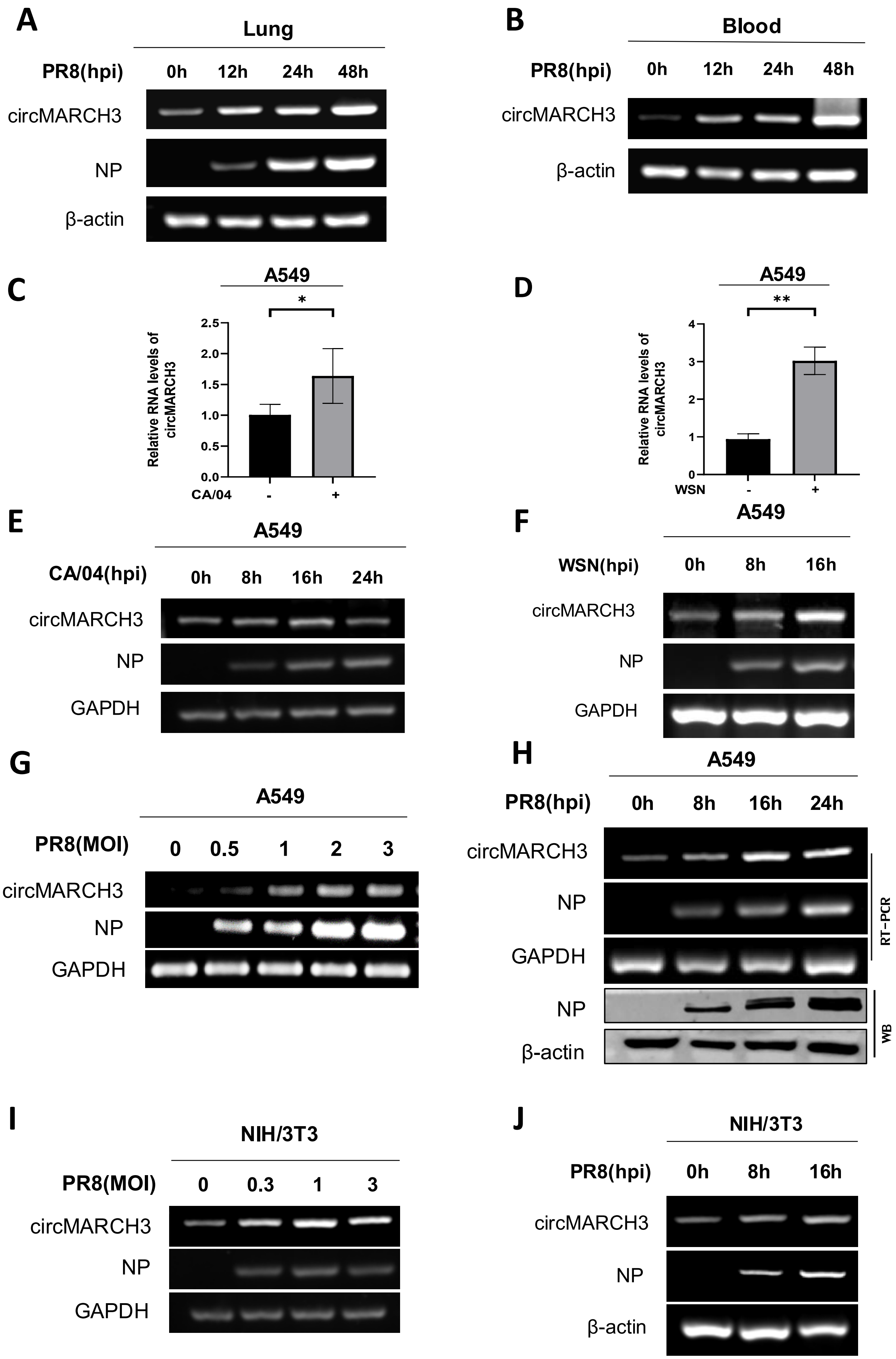
A
Lung
PR8(hpi) 0h 12h 24h 48h
circMARCH3
NP
β-actin
B
Blood
PR8(hpi) 0h 12h 24h 48h
circMARCH3
β-actin
C
A549
*
Relative RNA levels of circMARCH3
2.5
2.0
1.5
1.0
0.5
0.0
CA/04 - +
D
A549
**
Relative RNA levels of circMARCH3
4
3
2
1
0
WSN - +
E
A549
CA/04(hpi) 0h 8h 16h 24h
circMARCH3
NP
GAPDH
F
A549
WSN(hpi) 0h 8h 16h
circMARCH3
NP
GAPDH
G
A549
PR8(MOI) 0 0.5 1 2 3
circMARCH3
NP
GAPDH
H
A549
PR8(hpi) 0h 8h 16h 24h
circMARCH3
NP
GAPDH
RT-PCR
NP
β-actin
WB
I
NIH/3T3
PR8(MOI) 0 0.3 1 3
circMARCH3
NP
GAPDH
J
NIH/3T3
PR8(hpi) 0h 8h 16h
circMARCH3
NP
β-actin

Figure S3

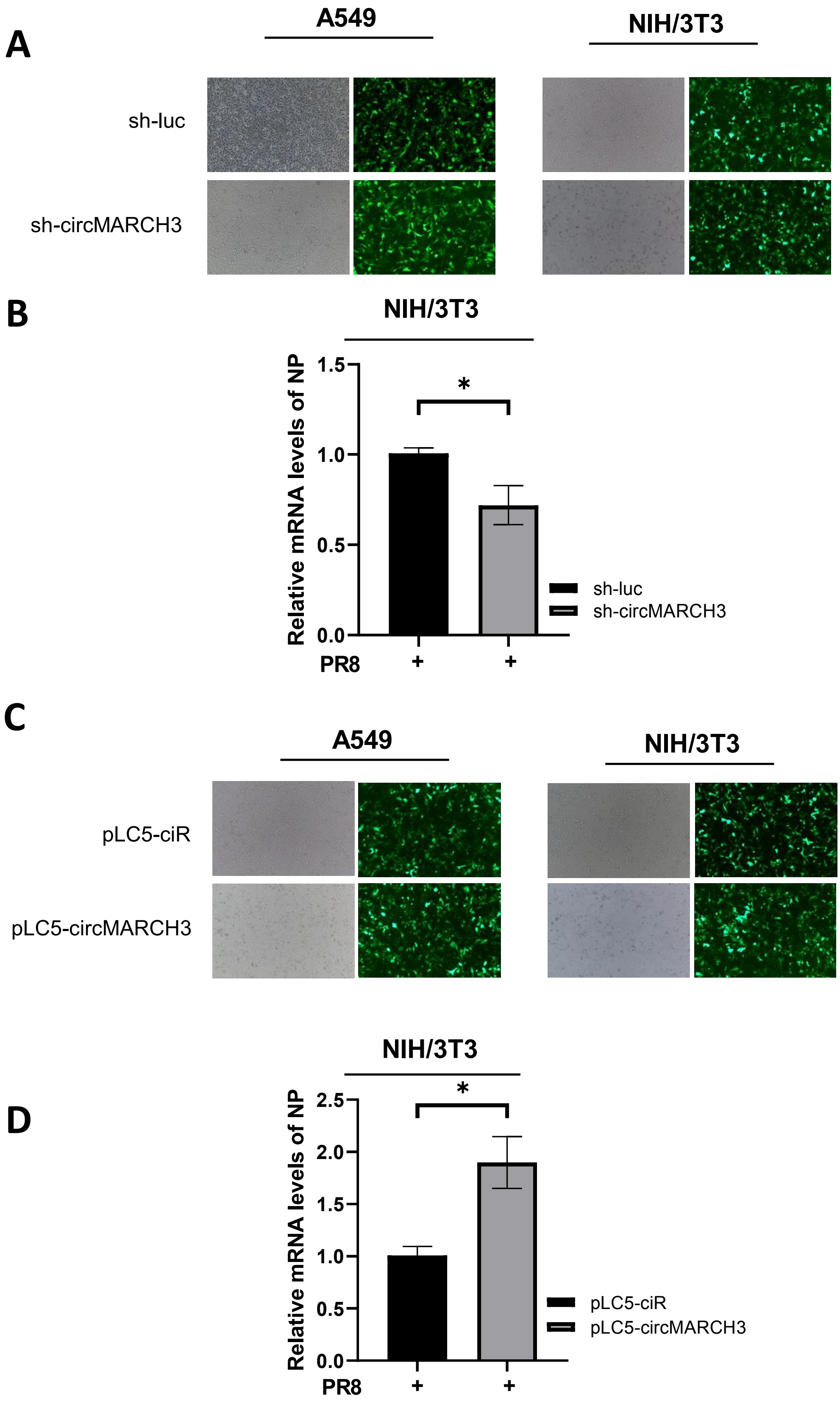
A
A549
NIH/3T3
sh-luc
sh-circMARCH3
B
NIH/3T3
Relative mRNA levels of NP
1.5
1.0
0.5
0.0
*
sh-luc
sh-circMARCH3
PR8
+
+
C
A549
NIH/3T3
pLC5-ciR
pLC5-circMARCH3
D
NIH/3T3
Relative mRNA levels of NP
2.5
2.0
1.5
1.0
0.5
0.0
*
pLC5-ciR
pLC5-circMARCH3
PR8
+
+

Figure S4

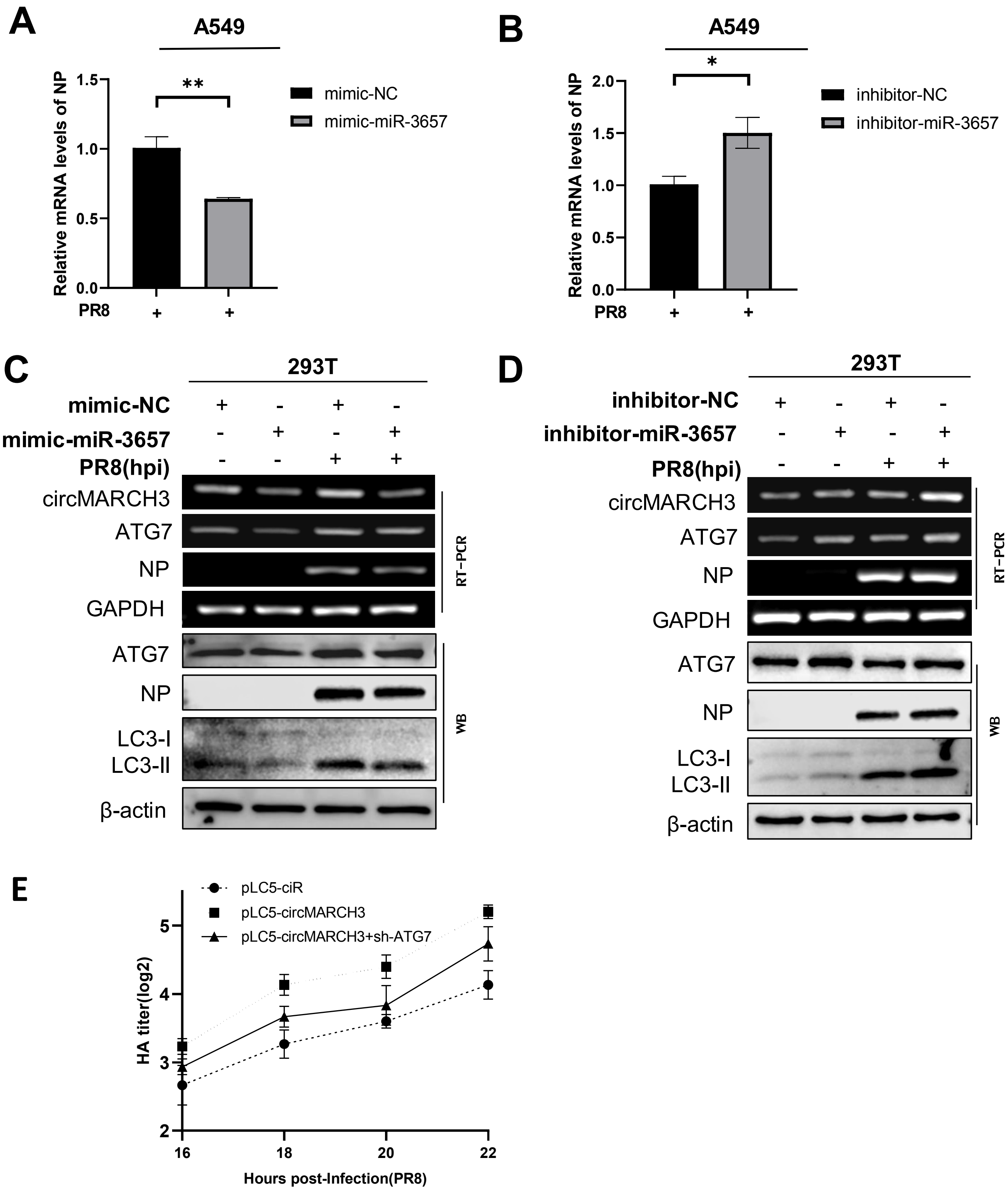

A
A549
**
Relative mRNA levels of NP
1.5
1.0
0.5
0.0
PR8
+
+
mimic-NC
mimic-miR-3657
B
A549
*
Relative mRNA levels of NP
2.0
1.5
1.0
0.5
0.0
PR8
+
+
inhibitor-NC
inhibitor-miR-3657
C
293T
mimic-NC
mimic-miR-3657
PR8(hpi)
circMARCH3
ATG7
NP
GAPDH
ATG7
NP
LC3-I
LC3-II
β-actin
RT−PCR
WB
D
293T
inhibitor-NC
inhibitor-miR-3657
PR8(hpi)
circMARCH3
ATG7
NP
GAPDH
ATG7
NP
LC3-I
LC3-II
β-actin
RT−PCR
WB
E
pLC5-ciR
pLC5-circMARCH3
pLC5-circMARCH3+sh-ATG7
HA titer(log2)
5
4
3
2
16
18
20
22
Hours post-Infection(PR8)

2 Supplementary Tables

Table S1 Primer sequences used in this study.

Primers	Sequence
miR-3657 (F)	GCGCGTGTGTCCCATTATTG
miR-3657 (R)	AGTGCAGGGTCCGAGGTATT
Sev-NP (F)	GCTTACGGGACAGATGAGAT
Sev-NP (R)	ATTGTTATGAACCGACTTGC
MDRV-P10 (F)	ATGGCTGACGCTTTTGAAGT
MDRV-P10 (R)	TAGTTAGATCTCGAGAGCCCG
HSV-UL30 (F)	ATCAACTTCGACTGGCCCTT
HSV-UL30 (R)	CCGTACATGTCGATGTTCAC
H9N2-NP (F)	CCTGCTTGTGTGTACGGACT
H9N2-NP (R)	TGAAGCAGACGGAAAGGGTC
H3N2-NP (F)	GAAGGGCGGTTGATCCAGAA
H3N2-NP (R)	TTCTTGGGATCTTTCCCCGC
VSV-NP (F)	GCAGAACACAAATGCCGAA
VSV-NP (R)	CGTTGCTAATGCGGCACAA
Human-GAPDH (F)	AGAAGGCTGGGGCTCATTTG
Human-GAPDH (R)	AGGGGCCATCCACAGTCTTC
ATG7 (F)	TTGACATGAGTGCTCCCACC
ATG7 (R)	AGACAGAGGGCAGGATAGCA
U6 (F)	CTCGCTTCGGCAGCACA

U6 (R)	AACGCTTCACGAATTTGCGT
PRV-gE (F)	CTTCCACTCGCAGCTCTTCT
PRV-gE (R)	TAGATGCAGGGCTCGTACAC
NP （PR8/WSN）(F)	TCAAACGTGGGATCAATG
NP （PR8/WSN）(R)	GTGCAGACCGTGCTAGAA
Mouse-β-actin (F)	AATGGGTCAGAAGGACTCCT
Mouse-β-actin (R)	ACGGTTGGCCTTAGGGTTCAG
Mouse-Mx1 (F)	AGTAAGTGCAGCTGGTTCCT
Mouse-Mx1 (R)	AAGGATCATTGCACAGCGAC
Human-Mx1 (F)	GACATTCGGCTGTTTACC
Human-Mx1 (R)	GCGGTTCTGTGGAGGTTA
Mouse-IFN-β (F)	GGTCCGAGCAGAGATCTTCA
Mouse-IFN-β (R)	CACTACCAGTCCCAGAGTCC
Human-IFN-β (F)	ACCAACAAGTGTCTCCTCCAA
Human-IFN-β (R)	TCTCCTCAGGGATGTCAAAGT
